# Antifungal Efficacy of Selenium Microparticles Biosynthesized by *Lysinibacillus sphaericus* Against Citrus Postharvest Blue and Green Molds: Mechanistic Insights

**DOI:** 10.3390/foods15183249

**Published:** 2026-09-14

**Authors:** Zeyu Xie, Sirong Lai, Siqi Guan, Mengxi Lv, Meng Zhang, Tingting Xiang, Runan Liao, Yingmei Tao, Zhenhua Jia, Yu Lei

**Affiliations:** 1 School of Food and Liquor Engineering, Sichuan University of Science and Engineering, Yibin 644000, China; 17883634588@163.com (Z.X.); 18140467795@163.com (S.G.); 13308190408@163.com (M.L.); zimo852@163.com (M.Z.); lrn17311789621@163.com (R.L.);; 2 YiBin Vocational and Technical College, Yibin 644100, China

**Keywords:** *Penicillium italicum*, *Penicillium digitatum*, selenite bioreduction, reactive oxygen species, membrane permeability

## Abstract

Citrus postharvest blue mold caused by *Penicillium italicum* and green mold caused by *P. digitatum* are major diseases responsible for fruit decay and economic losses. Increasing fungicide resistance and concerns regarding the safety of chemical fungicides have created an urgent need for alternative control strategies. In this study, selenium microparticles (SeMPs) were biosynthesized by *Lysinibacillus sphaericus* N30 and characterized by scanning electron microscopy coupled with energy-dispersive X-ray spectroscopy (SEM-EDS), X-ray diffraction (XRD), Raman spectroscopy, and dynamic light scattering (DLS). The control efficacy of N30-derived SeMPs against citrus postharvest diseases was evaluated using a 6-h post-inoculation treatment and a co-inoculation treatment, and their antifungal effects were further investigated by assessing reactive oxygen species (ROS) accumulation, membrane integrity, extracellular conductivity, and leakage of intracellular macromolecules. The N30-derived SeMPs were predominantly spherical and exhibited a Z-average hydrodynamic diameter of 656.5 nm with a PDI of 0.274. EDS identified Se as the predominant element, while XRD and Raman analyses supported the formation of predominantly amorphous elemental selenium. In vivo assays showed that 640 mg·L^−1^ SeMPs completely prevented blue mold incidence in the *P. italicum* co-inoculation treatment, whereas 1280 mg·L^−1^ completely suppressed blue mold under both application modes. Green mold caused by *P. digitatum* was completely suppressed at 1280–2560 mg·L^−1^, with co-inoculation treatment generally showing greater efficacy than post-inoculation treatment. SeMP exposure was associated with increased intracellular ROS accumulation, loss of membrane integrity, increased extracellular conductivity, and leakage of intracellular macromolecules in both pathogens. *P. italicum* showed greater susceptibility to SeMPs-associated membrane perturbation than *P. digitatum*, consistent with its higher sensitivity in the fruit assays. These findings indicate that biogenic SeMPs produced by *L. sphaericus* N30 have potential as selenium-based antifungal materials for the management of citrus postharvest blue and green molds.

## 1. Introduction

Citrus is one of the most widely cultivated, highest-yielding, and most extensively traded fruits globally, holding a central position in the world fruit industry [1]. Citrus comprises diverse species and varieties, including lemon, grapefruit, sweet orange, and mandarin, and is rich in vitamins, minerals, and various bioactive compounds, offering high nutritional and economic value [2]. China is a major citrus producer worldwide; according to the most recent available national statistics, the national citrus orchard area reached 3061.3 thousand hectares in 2023, accounting for 24.0% of the total orchard area, and citrus production reached 67.9149 million metric tons in 2024, representing approximately 20.0% of the national total fruit output. Among different citrus types, mandarins have the highest production, followed by sweet oranges, with grapefruit and pummelo lower, and lemon production relatively limited. Such substantial planting scale and output impose higher demands on citrus postharvest preservation, storage, and distribution; therefore, extending the postharvest storage life of citrus fruit and maintaining its commercial quality are of great significance for ensuring stable market supply and improving the economic benefits of the industry [3]. However, citrus fruit is highly susceptible to mechanical damage and pathogenic fungal infection during harvesting, transportation, and storage. Among these, blue mold caused by *Penicillium italicum* and green mold caused by *P. digitatum* are the most common and most destructive postharvest diseases, causing severe fruit decay and economic losses [4]. These two pathogens are widely distributed across major citrus-producing regions worldwide and have been detected in imported fruits in multiple countries [5]. Studies have shown that they are the dominant *Penicillium* pathogens responsible for postharvest decay of various citrus cultivars, with decay rates and disease incidence higher than those of other pathogens, and the economic losses they cause can account for up to 90% of total citrus postharvest losses [4,6].

Currently, chemical fungicides remain the primary means for controlling citrus postharvest blue and green mold; however, their prolonged or irrational use has led to varying degrees of resistance in pathogens such as *P. italicum* and *P. digitatum* against multiple mainstream fungicides, including benzimidazoles (e.g., thiabendazole), demethylation inhibitors (DMI; e.g., imazalil), and quinone outside inhibitors (QoI; e.g., azoxystrobin) [7]. Meanwhile, problems such as pesticide residues, environmental pollution, and food safety risks have become increasingly prominent. Therefore, developing efficient, safe, and environmentally friendly alternative technologies has become an urgent need in the field of citrus postharvest preservation [8].

Selenium is an essential trace element with important biological functions, although excessive exposure can cause toxicity [9]. Particulate forms of elemental selenium have attracted considerable attention because their physicochemical and biological properties can vary markedly with particle size, morphology, surface chemistry, and preparation route. Most previous antimicrobial studies have focused on selenium nanoparticles (SeNPs), which have shown inhibitory activity against a range of bacterial and fungal microorganisms. Proposed responses associated with SeNPs exposure include oxidative stress, alterations in membrane permeability, and cellular structural damage [10,11,12]. However, these findings should not be directly extrapolated to selenium particles outside the conventional nanoscale because particle size can influence surface area, dispersion behavior, and particle–cell interactions. According to the U.S. National Nanotechnology Initiative and ISO terminology, the nanoscale generally corresponds to approximately 1–100 nm [13,14]. Accordingly, selenium materials with substantially larger characteristic dimensions should be distinguished from conventional SeNPs.

Importantly, selenium microparticles (SeMPs) have also been reported in the literature and have demonstrated biological activity. Lu et al. incorporated SeMPs with an average particle size of approximately 900 nm into polylactic acid films and observed enhanced antibacterial and antioxidant properties [15]. Dat et al. prepared green-synthesized SeMPs and demonstrated antimicrobial activity of SeMP-containing composites against both bacterial and fungal microorganisms [16]. Similarly, Tuyen et al. explicitly classified spherical selenium particles with an average diameter of approximately 475 nm as SeMPs and reported antibacterial and antifungal activity [17]. These studies indicate that biologically active selenium particulate materials are not restricted to the conventional nanoparticle size range. Nevertheless, compared with SeNPs, the antimicrobial properties of SeMPs remain considerably less studied, particularly against postharvest phytopathogenic fungi.

Biological reduction in soluble selenium oxyanions provides an attractive route for producing elemental selenium particles. A variety of microorganisms can reduce selenite or selenate to insoluble elemental selenium, and the resulting particle characteristics depend strongly on the microbial strain, culture conditions, reaction time, and associated biomolecular coatings [18,19]. In our preliminary work, a selenite-tolerant strain, *Lysinibacillus sphaericus* N30, was isolated from selenium-rich soil and was found to efficiently reduce selenite and generate spherical selenium particles. Because characterization of the resulting material showed particle dimensions exceeding the conventional nanoscale, the N30-derived material is herein referred to as selenium microparticles (SeMPs).

Despite growing evidence for the antimicrobial activity of selenium-based particulate materials, little is known about the efficacy of SeMPs against the major postharvest citrus pathogens *P. italicum* and *P. digitatum*. In particular, their effective concentration range, performance under different post-inoculation application intervals, and associated cellular responses remain unclear. Therefore, in the present study, SeMPs were biosynthesized using *L. sphaericus* N30 and characterized for morphology, elemental composition, and particle-size distribution. Their antifungal activity against *P. italicum* and *P. digitatum* was evaluated both in vitro and on citrus fruit using a 6-h post-inoculation treatment and a co-inoculation treatment. Changes in hyphal morphology, membrane and cell-wall integrity, intracellular ROS accumulation, extracellular conductivity, and leakage of intracellular constituents were further examined to characterize the cellular responses associated with SeMPs exposure. This study provides a basis for evaluating the potential of bacterially derived selenium microparticles as candidate materials for citrus postharvest disease management.

## 2. Materials and Methods

### 2.1. Materials

#### 2.1.1. Microorganisms

Strain N30, used for the biosynthesis of selenium microparticles, was isolated from selenium-rich soil collected in Laojunshan, Pingshan County, Yibin City, Sichuan Province, China, and exhibited high tolerance to sodium selenite. Based on 16S rRNA gene sequence analysis and phylogenetic reconstruction, strain N30 was identified as *Lysinibacillus sphaericus*. Its 16S rRNA gene sequence was deposited in GenBank under accession number PP053464. The fungal pathogens used in this study, *Penicillium italicum* GJPI002 and *P. digitatum* GJPD001, were previously isolated and maintained in our laboratory. Their taxonomic identities were confirmed by multilocus phylogenetic analysis based on concatenated ITS, BenA, CaM, and RPB2 sequences (Figure S1). The previous identification and characterization of these two fungal strains have been reported in our earlier studies [20,21]. Both fungal strains were revived on potato dextrose agar (PDA) before use.

#### 2.1.2. Citrus Fruit

The tested Orah mandarin (*Citrus reticulata* cv. Orah) fruits were harvested from a citrus orchard in Yibin City, Sichuan Province, China, and transported to the laboratory within 2 h after harvest. Fruit selection and surface disinfection were performed according to Wang et al., with minor modifications [22]. Healthy fruits with uniform peel color, consistent maturity, similar size, intact surfaces, and no mechanical damage, pest infestation, or visible lesions were selected. The fruits were first rinsed with tap water to remove surface debris and then surface-disinfected by immersion in 2% (*v*/*v*) sodium hypochlorite for 2 min. Subsequently, the fruits were rinsed thoroughly three times with sterile water and air-dried at room temperature before use.

#### 2.1.3. Chemicals and Reagents

Sodium selenite (Na_2_SeO_3_, 99% purity) was obtained from Adamas Reagent Co., Ltd. (Shanghai, China). The SYTOX Green dead-cell fluorescent stain and propidium iodide (PI) staining kit were purchased from KeyGen Biotech (Jiangsu, China). Calcofluor White (CFW) was obtained from Solarbio (Beijing, China), and 2′,7′-dichlorodihydrofluorescein diacetate (DCFH-DA) was obtained from Aladdin (Shanghai, China). Glutaraldehyde (2.5%) was obtained from Macklin (Shanghai, China), and phosphate-buffered saline (PBS, pH 7.2–7.4) was obtained from Labgic (Beijing, China).

### 2.2. Biosynthesis and Preparation of SeMPs

#### 2.2.1. Biosynthesis of SeMPs by *L. sphaericus* N30

SeMPs were biosynthesized by *L. sphaericus* N30 according to the bacterial selenite-reduction method described by Bulgarini et al., with modifications [19]. *L. sphaericus* N30 was activated on Luria–Bertani (LB) agar medium, and a single colony was inoculated into LB broth and cultured at 35 °C and 180 rpm in a constant-temperature shaking incubator (ZWY-100H, Zhicheng, Shanghai, China) until the culture reached an OD_600_ of 0.8–1.0. The resulting culture was used as the seed culture and inoculated at 3% (*v*/*v*) into fresh LB broth supplemented with 5 mmol·L^−1^ sodium selenite. The cultures were subsequently incubated at 35 °C and 180 rpm for 3 d to facilitate bacterial reduction in selenite and the biosynthesis of SeMPs.

#### 2.2.2. Purification and Storage of SeMPs

Biogenic SeMPs were extracted and purified according to the method described by Shakibaie et al., with modifications [11]. After cultivation, the biomass was collected by centrifugation at 4 °C and 8000 rpm for 10 min using a TGL-21M benchtop high-speed refrigerated centrifuge (Luxiangyi, Shanghai, China; maximum radius, Rmax ≈ 9.4 cm), washed three times with sterile water, subjected to three freeze–thaw cycles, and resuspended in sterile water. The suspension was sonicated on ice at 100 W using a 5 s on/5 s off cycle for a total of 10 min. The disrupted cells were washed three times with 1.5 mol·L^−1^ Tris–HCl buffer (pH 8.3) containing 1% (*w*/*v*) sodium dodecyl sulfate (SDS), followed by three washes with sterile water. The pellet was resuspended in sterile water, mixed with n-octanol at a ratio of 1:1 (*v*/*v*), vortexed, and centrifuged at 2000 rpm for 5 min. The mixture was then allowed to stand at 4 °C for 24 h for phase separation. The upper and interfacial layers containing cell debris were discarded, and the lower aqueous phase containing SeMPs was collected and washed sequentially with chloroform, ethanol, and sterile water, with centrifugation at 4000 rpm for 10 min to collect the pellet. The obtained SeMPs were thoroughly dialyzed using a regenerated cellulose membrane with a molecular weight cut-off of 6–8 kDa to remove residual SDS and free selenite. The purified SeMPs were pre-frozen at −80 °C, freeze-dried, and stored at 4 °C protected from light until use.

#### 2.2.3. Preparation of SeMP Stock Solutions

After purification and freeze-drying, a known mass of the dried SeMPs preparation was accurately weighed using an analytical balance and dispersed in a defined volume of distilled water. The concentration of the SeMP suspension was expressed on a dry-mass basis and calculated as C = m/V (mg·L^−1^), where m is the dry mass of the freeze-dried SeMP preparation and V is the final volume of the suspension. Therefore, the concentrations reported in this study represent the dry-mass concentrations of the purified SeMP preparation rather than the concentration of elemental selenium. For each experiment, a 25,600 mg·L^−1^ SeMP stock suspension was freshly prepared and sonicated at 40 W for 5 min, followed by twofold serial dilution to obtain suspensions of 12,800, 6400, and 3200 mg·L^−1^ as required. The freshly prepared suspensions were kept at 4 °C in the dark only during the short interval before experimental use and were used on the same day. No prepared SeMP suspension was stored for subsequent experimental use. The freeze-dried SeMP preparation, rather than the aqueous suspension, was stored at 4 °C protected from light until use.

### 2.3. Preparation of Pathogen Spore and Mycelial Suspensions

#### 2.3.1. Preparation of Spore Suspensions

Spore suspensions of *P. digitatum* and *P. italicum* were prepared according to the method described in previous studies, with modifications [21]. *P. digitatum* and *P. italicum* were separately inoculated onto potato dextrose agar (PDA) plates and incubated at 25 °C for approximately 7 d until the colonies fully covered the plates and abundant spores were produced. A diluted potato dextrose broth (PDB) medium containing 5% (*v*/*v*) standard-strength PDB was prepared by mixing standard-strength PDB with sterile distilled water at a volume ratio of 5:95. Agar plugs containing abundant spores were excised from the cultures and transferred into the 5% (*v*/*v*) PDB medium, followed by agitation to release the spores. The resulting spore suspensions were filtered through three layers of sterile lens paper to remove mycelial fragments. Spore concentrations were determined using a hemocytometer and adjusted to 1 × 10^6^ spores·mL^−1^ with the same 5% (*v*/*v*) PDB medium. The prepared spore suspensions were stored at 4 °C until use.

#### 2.3.2. Mycelial Suspension

One milliliter of each spore suspension prepared as described in Section 2.3.1 was inoculated into 9 mL of potato dextrose broth (PDB). The cultures were incubated separately at 25 °C and 160 rpm for 48 h to obtain actively growing mycelia. The resulting cultures containing mycelia were used as the mycelial suspensions for subsequent experiments.

### 2.4. Characterization of the Selenite Reduction System and SeMPs

#### 2.4.1. SEM Observation of N30 Cells and the Selenite Reduction System

To observe cell morphology and SeMP deposition during selenite reduction by N30, blank control and treatment groups were established. N30 seed culture (OD_600_ ≈ 0.8–1.0) was used. No Na_2_SeO_3_ was added to the blank control group, whereas the treatment group was inoculated as described in Section 2.2.1 into LB liquid medium containing 5 mmol·L^−1^ sodium selenite. Samples were collected after cultivation at 35 °C and 180 rpm for 72 h. Samples for SEM observation were prepared according to a previously described method, with modifications [20]. The samples were collected by centrifugation at 4 °C and 8000 rpm for 10 min, washed three times with sterile PBS (pH 7.4), and fixed with 2.5% glutaraldehyde prepared in PBS (pH 7.2–7.4) at 4 °C for 24 h. After dehydration through a graded ethanol series (50–95%) followed by absolute ethanol, critical-point drying, and sputter coating using an SBC-2 ion sputter coater (JS-19029, Beijing Zhongke Keyi, Beijing, China), the samples were observed and photographed using a scanning electron microscope (SEM; VEGA 3SBU, TESCAN, Brno, Czech Republic).

#### 2.4.2. Morphological, Elemental, and Particle Size Characterization of SeMPs

The morphological, elemental, structural, and particle-size characteristics of the SeMPs were analyzed with reference to previously reported characterization approaches for biogenic selenium particles [23]. SeMPs prepared as described in Section 2.2 were dispersed in ultrapure water, and 5 μL of the suspension was deposited onto carbon conductive adhesive and air-dried. After sputter coating using the same SBC-2 ion sputter coater, the surface morphology of the SeMPs was observed using the same scanning electron microscope (SEM), and their elemental composition was analyzed using a coupled Bruker energy-dispersive X-ray spectrometer (EDS).

For structural characterization, freeze-dried SeMPs were ground into a fine powder and passed through a 200-mesh sieve prior to analysis. The crystalline structure of the SeMPs was examined by X-ray diffraction (XRD) using a D2 PHASER diffractometer (Bruker AXS, Karlsruhe, Germany) equipped with a Cu target. Diffraction patterns were recorded over a 2θ range of 5–90°, with a step size of 0.02° and a counting time of 0.1 s per step. Raman spectroscopy was subsequently performed using a DXR3 Raman spectrometer (Thermo Fisher Scientific, Waltham, MA, USA) to characterize the vibrational features and structural state of the SeMPs.

Separately, SeMPs dispersed in ultrapure water were analyzed for particle-size distribution by dynamic light scattering (DLS) using a Zetasizer Nano ZS90 (Malvern Panalytical, Great Malvern, UK). The Z-average hydrodynamic diameter and polydispersity index (PDI) were recorded to characterize the hydrodynamic size distribution of the SeMP suspension.

### 2.5. Antifungal Activity of SeMPs

#### 2.5.1. In Vitro Antifungal Activity and Determination of MIC and MFC

The in vitro antifungal activity of SeMPs against *P. digitatum* and *P. italicum*, including the determination of minimum inhibitory concentration (MIC) and minimum fungicidal concentration (MFC), was evaluated using a broth microdilution method according to Jia et al., with modifications [24]. Spore suspensions of *P. digitatum* and *P. italicum* were prepared in 5% (*v*/*v*) PDB as described in Section 2.3.1. In a 96-well microplate, 180 μL of the spore suspension was added to each well, followed by 20 μL of SeMP suspension at concentrations of 50, 100, 200, 400, 800, 1600, 3200, or 6400 mg·L^−1^, resulting in final SeMP concentrations of 5, 10, 20, 40, 80, 160, 320, or 640 mg·L^−1^, respectively. For the untreated growth control (0 mg·L^−1^ SeMPs), 20 μL of sterile distilled water was added instead of the SeMP suspension. Background wells containing the corresponding concentrations of SeMPs but no spores were included to correct for particle-associated optical interference. After thorough mixing by pipetting, the microplates were incubated statically at 25 °C for 48 h. MIC determination: The OD_600_ values were measured using a microplate reader at 0 h and after 48 h of incubation. To minimize interference caused by the intrinsic optical properties of SeMPs, the OD_600_ values were corrected by subtracting the corresponding particle-background absorbance: corrected OD600 = (sample 48 h − sample 0 h) − (background 48 h − background 0 h). The mycelial growth inhibition rate at each SeMP concentration was calculated relative to the untreated growth control, which was defined as 100% growth. The MIC was defined as the lowest SeMP concentration that completely inhibited detectable fungal growth after 48 h of incubation. MFC determination: After 48 h of incubation, a 100-μL aliquot from each treatment well was evenly spread onto PDA plates and incubated at 25 °C for 48 h. The MFC was defined as the lowest SeMP concentration at which no fungal colonies were observed on the PDA plates.

#### 2.5.2. Control Efficacy of SeMPs Against Citrus Postharvest Blue and Green Molds

Two treatment modes were established according to the interval between pathogen inoculation and SeMP application: post-inoculation treatment, in which SeMPs were applied 6 h after pathogen inoculation, and a co-inoculation treatment, in which SeMPs were applied immediately after pathogen inoculation. Pathogen-inoculated fruits treated with distilled water served as the corresponding controls.

The fruit inoculation and disease-control assay was performed based on the method of Ibrahim et al., with slight modifications [25]. A wound approximately 2 mm deep was made near the equator of each fruit, and 10 μL of the spore suspension of *P. digitatum* or *P. italicum* was inoculated into each wound. For SeMP treatment, each fruit was completely immersed in a SeMP suspension at 320, 640, 1280, or 2560 mg·L^−1^, maintained in the fully immersed state for 1 s, and then immediately removed. Control fruits were treated in the same manner with distilled water and designated as the 0 mg·L^−1^ treatment.

For the 6-h post-inoculation treatment, inoculated fruits were maintained at 25 ± 1 °C and 90 ± 5% relative humidity for 6 h and then treated with the corresponding SeMP suspension using the immersion procedure described above. The fruits were air-dried and placed in preservation boxes for subsequent storage. For the co-inoculation treatment, the fruits were treated with the corresponding SeMP suspension immediately after pathogen inoculation using the same immersion procedure, air-dried, and placed in preservation boxes. All fruits were subsequently stored at 25 ± 1 °C and 90 ± 5% relative humidity. Disease incidence and lesion diameter were recorded approximately 10 d after treatment, when the untreated controls were fully diseased. Each treatment consisted of three independent replicates, with nine fruits per replicate (27 fruits in total).

### 2.6. Effects of SeMPs on Fungal Cell Structure and Physiology

#### 2.6.1. Effect of SeMPs on Hyphal Surface Morphology

The method of Sun et al. [26] was followed with slight modifications. Mycelial suspension was prepared as described in Section 2.3.2. The mycelia were collected, washed three times with sterile water, and resuspended to the original volume. Nine hundred microliters of mycelial suspension was mixed with 100 μL of SeMP stock solution to obtain final SeMP concentrations of 320 and 2560 mg·L^−1^; the control received an equal volume of distilled water. After incubation at 25 °C for 24 h, the mycelia were collected by centrifugation at 4000 rpm for 10 min. Fixation and dehydration were performed as described for N30 cells in Section 2.4.1.

#### 2.6.2. Effect of SeMPs on Cell Membrane Integrity

The method of Wang et al. [27] was followed with slight modifications. Mycelial suspension was prepared as described in Section 2.3.2. One hundred eighty microliters of mycelial suspension was mixed with 20 μL of SeMP stock solution to obtain final SeMP concentrations of 320, 640, 1280, and 2560 mg·L^−1^; the control received an equal volume of sterile water. After incubation for 6 h, the mycelia were collected by centrifugation at 4 °C and 6000 rpm for 5 min, washed three times with PBS, and resuspended to the original volume. SYTOX Green was added to a final concentration of 1 μmol·L^−1^, and the samples were incubated at room temperature in the dark for 5 min. The samples were then washed with PBS to remove excess dye and observed and photographed using a fluorescence microscope.

#### 2.6.3. Effect of SeMPs on Fungal Cell Wall Integrity

Mycelial suspensions were prepared as described in Section 2.3.2, and the SeMP treatment concentrations were the same as those described in Section 2.6.2. After incubation at 25 °C with shaking for 6 h, the mycelia were collected by centrifugation at 4000 rpm for 10 min, washed three times with PBS, and resuspended. Fungal cell wall integrity was evaluated using a Calcofluor White (CFW) Fungal Fluorescence Staining Kit (G3235, Beijing Solarbio Science & Technology Co., Ltd., Beijing, China) according to the manufacturer’s instructions, with minor modifications. Briefly, an appropriate amount of the mycelial suspension was placed on a microscope slide and air-dried. Subsequently, 20–50 μL of freshly prepared CFW working solution was added to the sample, followed by incubation in the dark for 0.5–1 min. The stained samples were directly observed without washing using a fluorescence microscope at an excitation wavelength of 355 nm and an emission wavelength of 440 nm, and representative images were captured to evaluate changes in fungal cell wall integrity.

#### 2.6.4. Effect of SeMPs on Intracellular Reactive Oxygen Species Levels

The method of Liu et al. [28] was followed with slight modifications. Mycelial suspension was prepared as described in Section 2.3.2, and the SeMP treatment concentrations were the same as those described in Section 2.6.2. After incubation at 25 °C with shaking for 6 h, the mycelia were collected by centrifugation at 4000 rpm for 10 min, washed three times with PBS, and resuspended. DCFH-DA was added to a final concentration of 10 μM, and the samples were incubated at 30 °C in the dark for 60 min. Unbound probe was removed by washing with PBS, and the samples were subsequently observed and photographed using a fluorescence microscope to evaluate intracellular reactive oxygen species (ROS) levels after SeMP treatment.

#### 2.6.5. Effect of SeMPs on Extracellular Conductivity

The method of Wang et al. [29] was followed with slight modifications. One hundred microliters of *P. digitatum* or *P. italicum* spore suspension was inoculated into 20 mL of PDB and cultured at 25 °C with shaking for 48 h. The mycelia were collected by centrifugation at 4000 rpm for 10 min, washed three times with PBS, and resuspended in 18 mL of sterile water. Subsequently, 2 mL of SeMP stock solution was added to obtain final SeMP concentrations of 320, 640, 1280, and 2560 mg·L^−1^; the control received an equal volume of sterile water. The cultures were further incubated at 25 °C with shaking. Samples were collected at 0, 1, 2, 3, 6, and 9 h after treatment and centrifuged at 4 °C and 6000 rpm for 2 min, after which the conductivity of the supernatant was measured.

#### 2.6.6. Effect of SeMPs on Nucleic Acid and Protein Leakage

The leakage of intracellular UV-absorbing constituents was assessed with modifications based on the method of Paul et al. [30]. Mycelial preparation and SeMP treatment were performed as described in Section 2.6.5. Samples were collected at 0, 1, 2, 3, 6, and 9 h after treatment and centrifuged at 4 °C and 10,000 rpm for 2 min to remove mycelia and suspended materials. The resulting supernatants were collected, and absorbance at 260 and 280 nm was measured using a NanoDrop ultramicro spectrophotometer(Thermo Fisher Scientific, Wilmington, DE, USA). Absorbance at 260 nm was used as an indicator of the release of nucleic-acid-associated intracellular constituents, whereas absorbance at 280 nm was used as an indicator of the release of protein-associated UV-absorbing constituents. Increased absorbance at these wavelengths was interpreted as evidence of enhanced leakage of intracellular components associated with increased membrane permeability.

### 2.7. Statistical Analysis

Data were organized using WPS Office, figures were prepared using Origin 2021, and statistical analyses were performed using IBM SPSS Statistics 26.0. Disease incidence, lesion diameter, and cell membrane integrity were analyzed by one-way analysis of variance (one-way ANOVA). When the ANOVA indicated a significant difference, Duncan’s new multiple range test was used for post hoc multiple comparisons. Extracellular conductivity and nucleic acid and protein leakage were analyzed by two-way ANOVA. When a significant interaction was detected, simple-effects analysis was performed, followed by Duncan’s new multiple range test for post hoc multiple comparisons. A value of *p* < 0.05 was considered statistically significant. All experiments were independently repeated three times, and the results were expressed as mean values ± SD (standard deviation).

## 3. Results

### 3.1. Characterization of SeMPs by SEM–EDS, XRD, Raman Spectroscopy, and Particle Size Analysis

**Figure 1:**
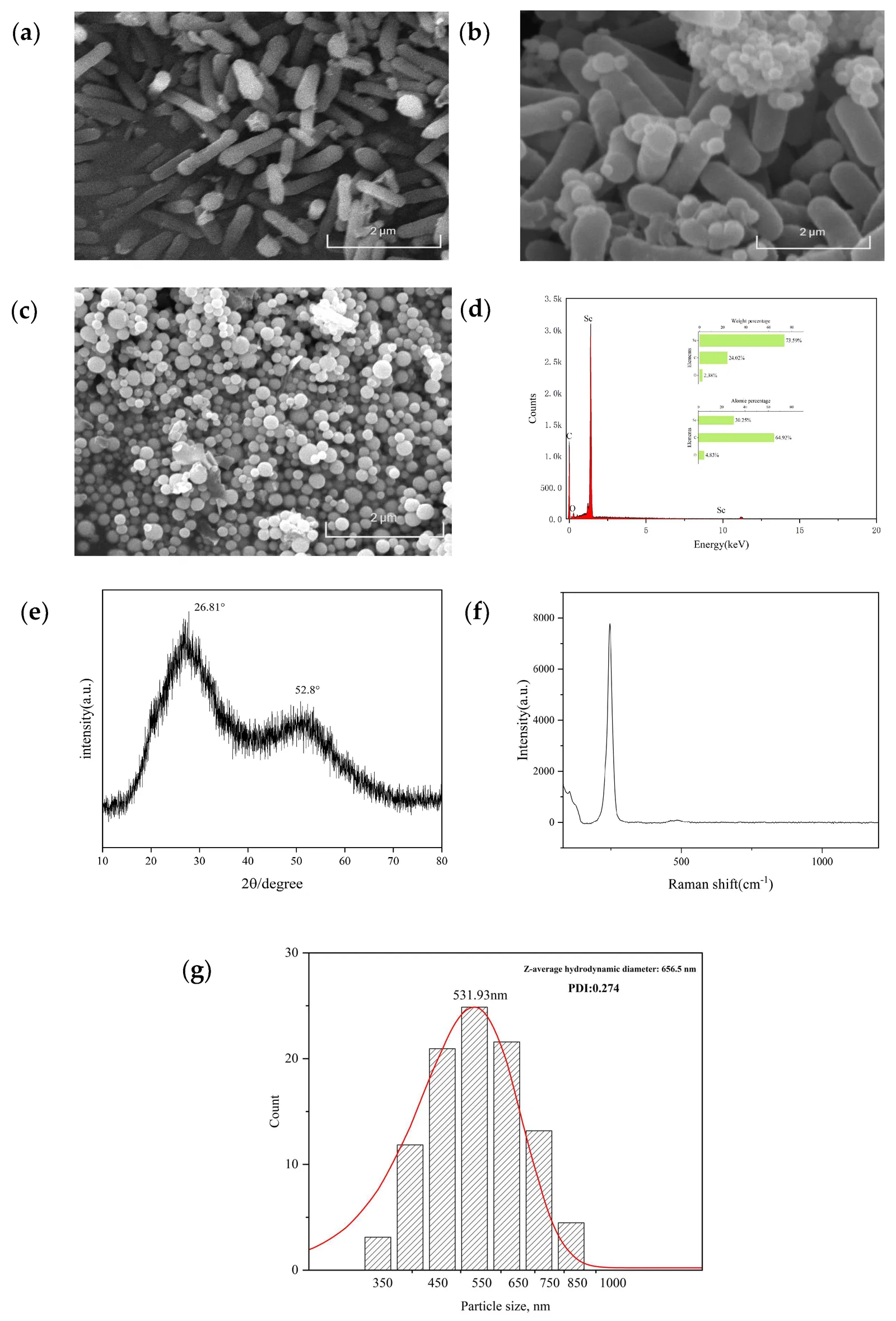
Morphological, elemental, structural, and size characterization of SeMPs biosynthesized by *L. sphaericus* N30. (**a**) SEM image of *L. sphaericus* N30 cells; (**b**) SEM image showing SeMPs associated with the cell surface; (**c**) SEM image of purified SeMPs; (**d**) EDS spectrum and elemental composition of purified SeMPs; (**e**) X-ray diffraction (XRD) pattern of purified SeMPs; (**f**) Raman spectrum of purified SeMPs; (**g**) particle-size distribution of purified SeMPs determined by dynamic light scattering (DLS).

As shown in Figure 1, the morphology, elemental composition, structural characteristics, and particle-size distribution of the selenium microparticles (SeMPs) biosynthesized by *L. sphaericus* N30 were characterized. SEM observations showed that strain N30 exhibited a typical rod-shaped morphology (Figure 1a). Following cultivation in the presence of sodium selenite, numerous particulate deposits were observed in association with the bacterial cell surface (Figure 1b). After purification, the recovered particles were predominantly spherical (Figure 1c).

EDS analysis showed that Se was the predominant element in the purified particles, accounting for 73.59% of the detected mass, followed by C (24.02%) and O (2.38%) (Figure 1d). Characteristic Se signals were observed at approximately 1.40 and 11.22 keV, confirming the enrichment of selenium in the purified particulate material. XRD analysis revealed broad diffraction features centered at approximately 26.81° and 52.80° (2θ), without distinct sharp Bragg reflections, indicating that the N30-derived SeMPs possessed a predominantly amorphous or poorly crystalline structure (Figure 1e). Consistently, Raman spectroscopy showed a dominant band in the 240 cm^−1^ region, corresponding to characteristic Se–Se vibrational modes associated with amorphous selenium (Figure 1f). Together with the EDS results, the XRD and Raman data support the formation of predominantly amorphous elemental selenium by *L. sphaericus* N30.

DLS analysis further showed that the purified SeMP suspension exhibited a size distribution mainly centered at approximately 531.93 nm, with a Z-average hydrodynamic diameter of 656.5 nm and a PDI of 0.274 (Figure 1g). The PDI value indicated a relatively narrow-to-moderate hydrodynamic size distribution. Based on the measured hydrodynamic diameter, the N30-derived selenium particles were classified as SeMPs rather than conventional selenium nanoparticles.

### 3.2. Growth Inhibition of Citrus Pathogens by SeMPs and MIC/MFC Analysis

**Figure 2:**
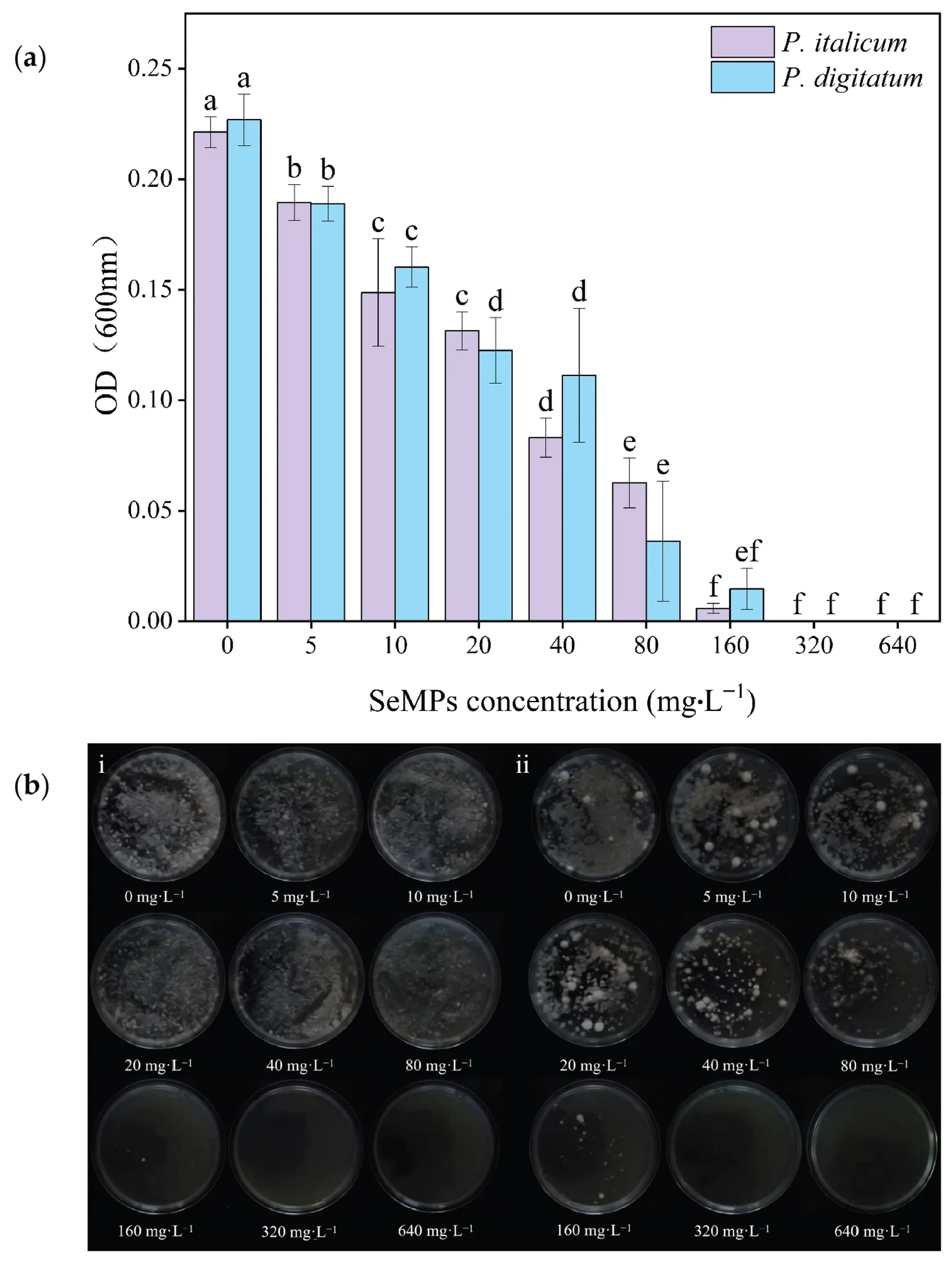
Growth inhibition and survival of *P. italicum* and *P. digitatum* treated with different concentrations of SeMPs. (**a**) Growth responses of *P. italicum* and *P. digitatum* to different SeMP concentrations; (**b**) fungal survival on PDA plates after SeMP treatment: (i) *P. italicum* and (ii) *P. digitatum*. Bars sharing the same letter are not significantly different.

As shown in Figure 2, the growth inhibition and survival of *P. digitatum* and *P. italicum* following treatment with different concentrations of SeMPs were evaluated. The OD_600_ measurements showed that SeMPs exerted a clear concentration-dependent inhibitory effect on both pathogens (Figure 2a). As the SeMP concentration increased, the OD_600_ values of both fungi gradually decreased, indicating progressively enhanced inhibition of mycelial growth. When the SeMP concentration reached 320 mg·L^−1^, the OD_600_ values of both pathogens decreased to nearly zero, and mycelial growth was completely inhibited. Accordingly, the minimum inhibitory concentration (MIC) of SeMPs against both *P. digitatum* and *P. italicum* was determined to be 320 mg·L^−1^.

The survival of the two pathogens after SeMP treatment was further evaluated using a PDA recovery assay (Figure 2b). The results showed that both pathogens still formed numerous colonies after treatment with low concentrations of SeMPs, whereas the number of colonies gradually decreased with increasing SeMP concentration. Viable colonies were still observed after treatment with 160 mg·L^−1^ SeMPs, whereas no colony growth was detected at 320 or 640 mg·L^−1^. Therefore, the minimum fungicidal concentration (MFC) of SeMPs against both pathogens was determined to be 320 mg·L^−1^. The identical MIC and MFC values further indicated that, at 320 mg·L^−1^, SeMPs not only completely inhibited the growth of *P. digitatum* and *P. italicum* but also prevented their subsequent recovery on PDA, demonstrating a fungicidal effect against both pathogens.

### 3.3. In Vivo Control Efficacy

**Figure 3:**
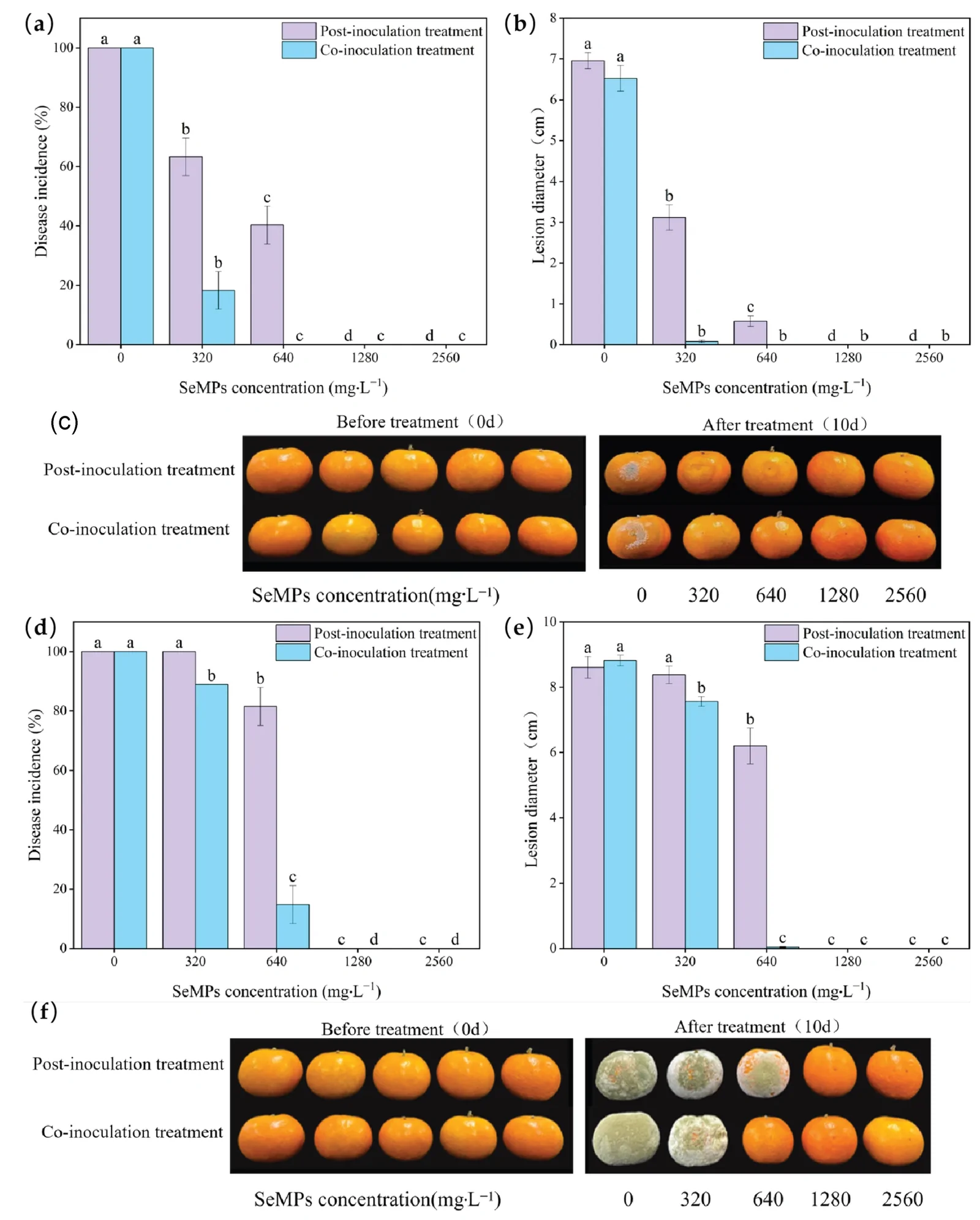
Control of postharvest blue mold and green mold in citrus fruit by SeMPs. (**a**–**c**) Fruit inoculated with *P. italicum*: (**a**) disease incidence, (**b**) lesion diameter, and (**c**) representative images of infected fruit. (**d**–**f**) Fruit inoculated with *P. digitatum*: (**d**) disease incidence, (**e**) lesion diameter, and (**f**) representative images of infected fruit. Post-inoculation treatment: SeMPs applied 6 h after pathogen inoculation. Co-inoculation: SeMPs applied simultaneously with the pathogen.Different lowercase letters above the bars indicate significant differences among treatments within the same panel according to Duncan’s new multiple range test (*p* < 0.05). Bars sharing the same letter are not significantly different.

As shown in Figure 3, SeMPs significantly inhibited the development of postharvest blue mold and green mold on citrus fruit, and the control efficacy was generally concentration-dependent. At 10 d after inoculation, the disease incidence of the control groups for both pathogens was 100 ± 0%. For *P. italicum*, the disease incidence under post-inoculation treatment was 63.3 ± 6.35% and 40.3 ± 6.35% at 320 and 640 mg·L^−1^, respectively, whereas under co-inoculation treatment it decreased to 18.3 ± 6.35% and 0%; at 1280 mg·L^−1^, both treatments completely inhibited disease occurrence (Figure 3a). For *P. digitatum*, the disease incidence under post-inoculation treatment at 320 mg·L^−1^ showed no significant difference from the control, whereas co-inoculation treatment reduced it to 88.9 ± 0% and 14.8 ± 6.41% at 320 and 640 mg·L^−1^, respectively. Both treatments completely prevented green mold occurrence at 1280 mg·L^−1^ and above (Figure 3d).

Changes in lesion diameter were consistent with the disease incidence results (Figure 3b,e). For *P. italicum*, post-inoculation treatment at 320 and 640 mg·L^−1^ reduced lesion diameter to 3.12 ± 0.20 and 0.578 ± 0.13 cm, respectively, whereas co-inoculation at 320 mg·L^−1^ already reduced lesion diameter to nearly 0 cm. For *P. digitatum*, the 320 mg·L^−1^ post-inoculation treatment did not significantly reduce lesion diameter compared with the untreated control, whereas increasing the concentration to 640 mg·L^−1^ reduced the lesion diameter to 6.20 ± 0.55 cm. In contrast, lesion development was almost completely suppressed under co-inoculation at 640 mg·L^−1^. No measurable lesions formed at 1280 mg·L^−1^ and above.

Fruit phenotypes were consistent with the above quantitative results (Figure 3c,f). Fruits without SeMP treatment exhibited extensive visible decay and mycelial coverage, whereas increasing SeMP concentrations progressively reduced lesion development and visible fungal growth. At the same concentration, co-inoculation treatment generally exhibited milder disease symptoms, especially within the medium concentration range; at high SeMP concentrations, no visible lesions appeared on the fruit. Overall, SeMPs effectively controlled postharvest blue and green mold on citrus fruit by reducing disease incidence and limiting lesion expansion, with a clear concentration dependence and pathogen-specific difference.

The two pathogens differed in their sensitivity to SeMPs. *P. italicum* was relatively more sensitive to SeMP treatment, particularly under co-inoculation treatment. At 320 mg·L^−1^, co-inoculation treatment reduced lesion diameter to nearly zero, and disease incidence further decreased to 0% at 640 mg·L^−1^. In contrast, *P. digitatum* showed lower sensitivity to SeMPs, especially under the 6-h post-inoculation treatment. At 640 mg·L^−1^, substantial disease incidence and lesion development remained under post-inoculation treatment, whereas co-inoculation treatment at the same concentration markedly suppressed both disease incidence and lesion expansion. At 1280 mg·L^−1^ and above, no visible disease development was observed for either pathogen under either treatment during the 10-d observation period. Overall, co-inoculation treatment generally provided greater disease suppression than the post-inoculation treatment, particularly at intermediate SeMP concentrations. These results indicate that the control efficacy of SeMPs was influenced by application timing and suggest that application at the time of pathogen inoculation may provide more effective early-stage disease control than application 6 h after inoculation.

### 3.4. Effect of SeMP Treatment on Mycelial Surface Morphology

**Figure 4:**
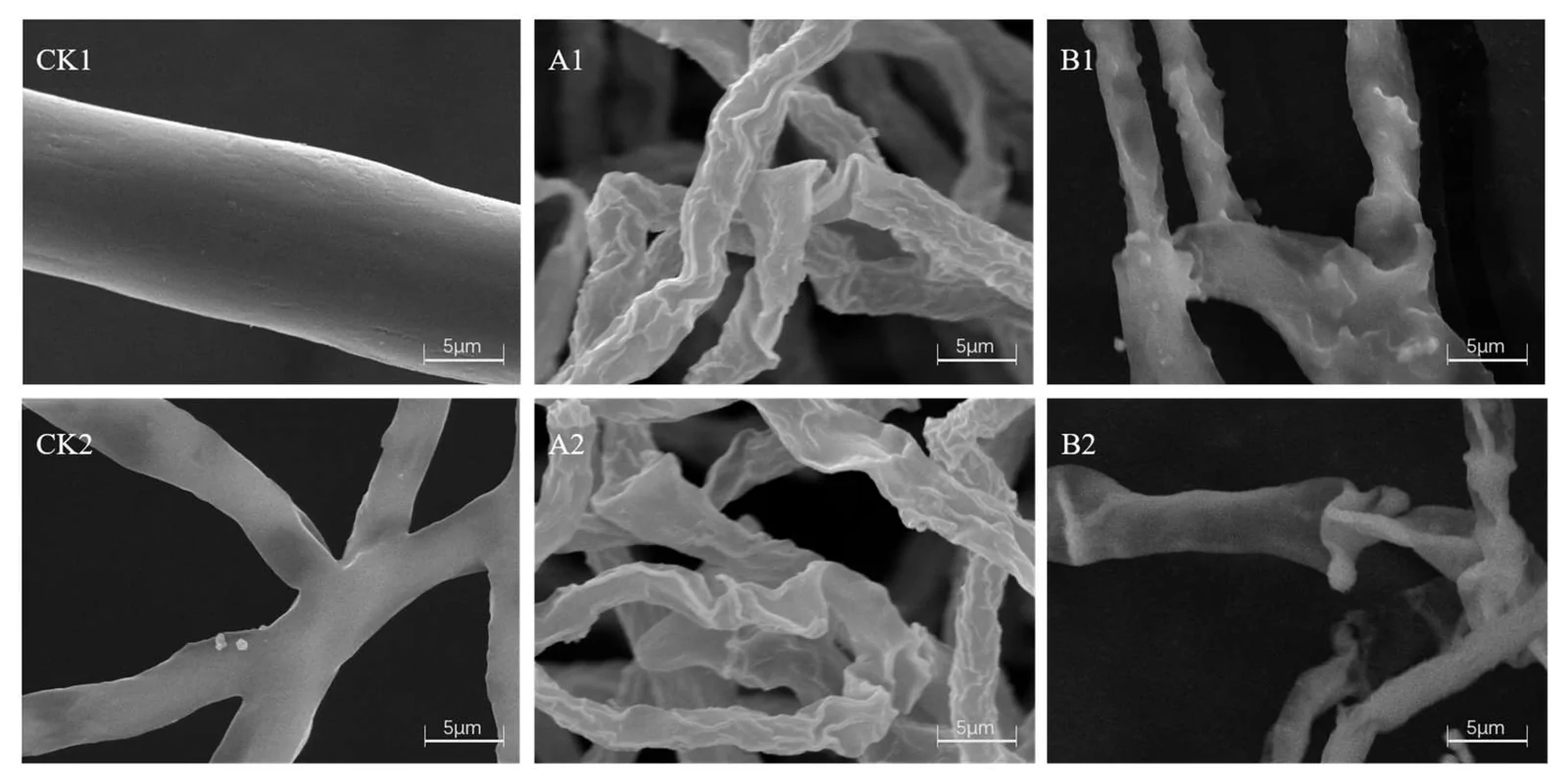
Effects of SeMP concentration on the surface morphology of *P. italicum* and *P. digitatum* hyphae. CK1, A1, and B1: *P. italicum* treated with 0, 320, and 2560 mg·L^−1^ SeMPs, respectively; CK2, A2, and B2: *P. digitatum* treated with 0, 320, and 2560 mg·L^−1^ SeMPs, respectively. Scale bar = 5 µm.

The effects of different SeMNP concentrations on the surface morphology of *P. italicum* and *P. digitatum* mycelia were observed by SEM. As shown in Figure 4, the untreated mycelia had smooth surfaces, full morphology, and no visible shrinkage. At 320 mg·L^−1^ SeMPs, both mycelia showed obvious shrinkage; when the concentration increased to 2560 mg·L^−1^, mycelial shrinkage was further intensified, with nearly complete collapse and even local depressions. These results indicate that SeMPs can effectively disrupt mycelial morphology, and the disruptive effect increases with concentration in a concentration-dependent manner.

### 3.5. Effect of SeMP Treatment on Cell Membrane Integrity

**Figure 5:**
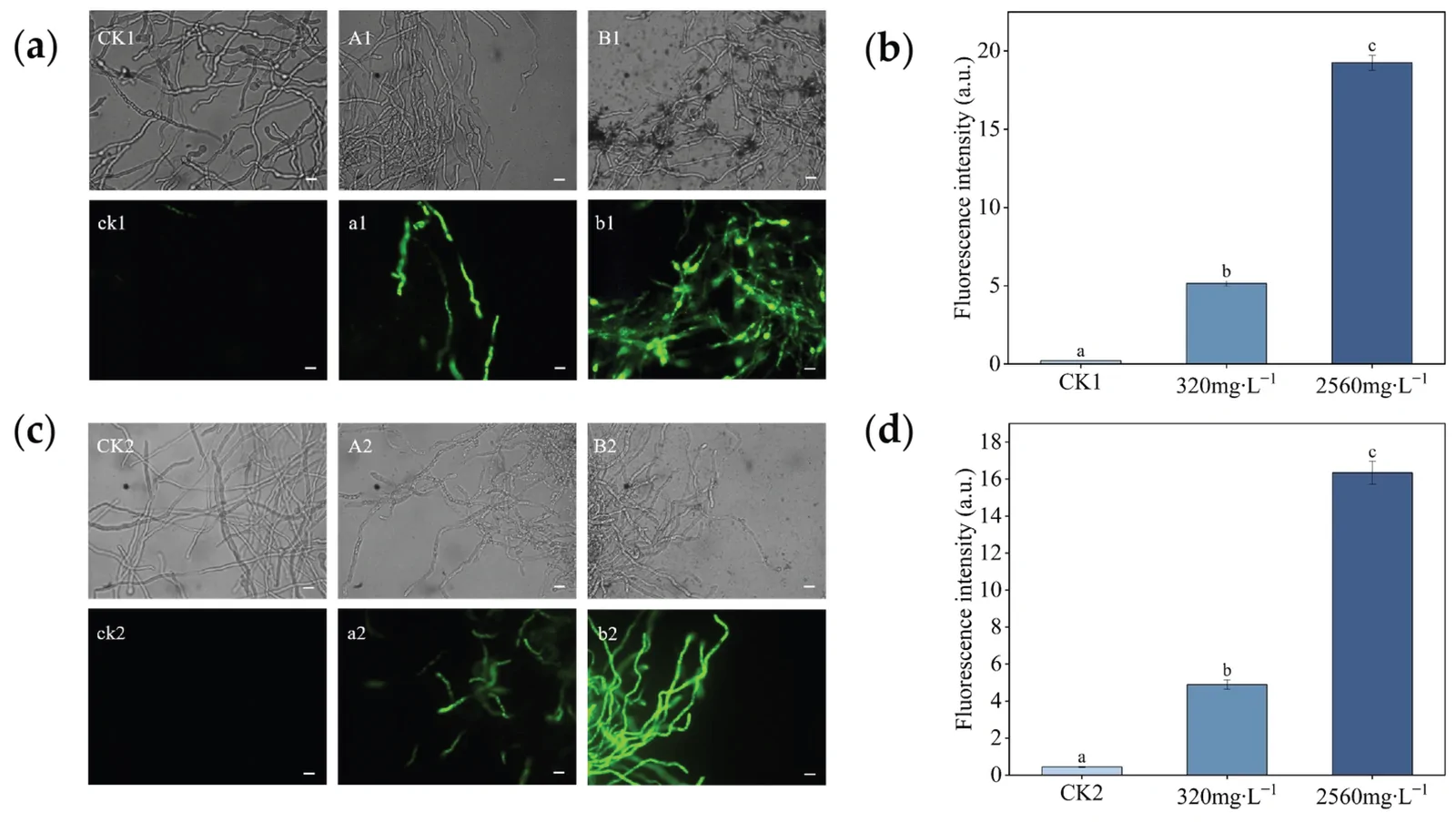
Effects of SeMP treatment on hyphal membrane integrity and fluorescence intensity in *P. italicum* and *P. digitatum*. (**a**,**c**) Bright-field and corresponding fluorescence images of *P. italicum* and *P. digitatum* hyphae treated with 0, 320, and 2560 mg·L^−1^ SeMPs, respectively; (**b**,**d**) quantitative analysis of hyphal membrane fluorescence intensity in the two fungi. Different lowercase letters above the bars indicate significant differences among treatments (*p* < 0.05). Scale bar = 100 μm.

To evaluate the effects of SeMPs on hyphal membrane integrity, SYTOX Green staining was employed to visualize fluorescence changes in *P. italicum* and *P. digitatum* hyphae following treatment with different concentrations of SeMPs. As shown in Figure 5a,c, little to no green fluorescence was observed in the hyphae of either pathogen in the control groups, indicating that their cell membranes remained largely intact. Following treatment with 320 mg·L^−1^ SeMPs, fragmented regions of intense fluorescence appeared in *P. italicum* hyphae, whereas fluorescence in *P. digitatum* was mainly localized to discrete regions and hyphal tips, suggesting that SeMPs had already induced varying degrees of membrane damage. When the SeMP concentration was increased to 2560 mg·L^−1^, the green fluorescence signals were markedly enhanced in both pathogens. Specifically, *P. italicum* hyphae exhibited dense, discontinuous, or punctate fluorescence, whereas *P. digitatum* hyphae displayed more continuous and intense fluorescence throughout the hyphae. Quantitative analysis of fluorescence intensity (Figure 5b,d) further showed that fluorescence intensity increased significantly with increasing SeMP concentration in both *P. italicum* and *P. digitatum*, with significant differences observed among the different treatment concentrations (*p* < 0.05). The highest fluorescence intensity was detected at 2560 mg·L^−1^, consistent with the fluorescence microscopy observations. These results indicate that SeMPs disrupt hyphal membrane integrity and increase membrane permeability in *P. italicum* and *P. digitatum* in a concentration-dependent manner, with more pronounced membrane damage occurring at the higher SeMP concentration. Consistent trends in membrane permeability were also observed at the intermediate SeMP concentrations of 640 and 1280 mg·L^−1^, as shown in Figure S2.

### 3.6. Effect of SeMP Treatment on the Mycelial Cell Wall

**Figure 6:**
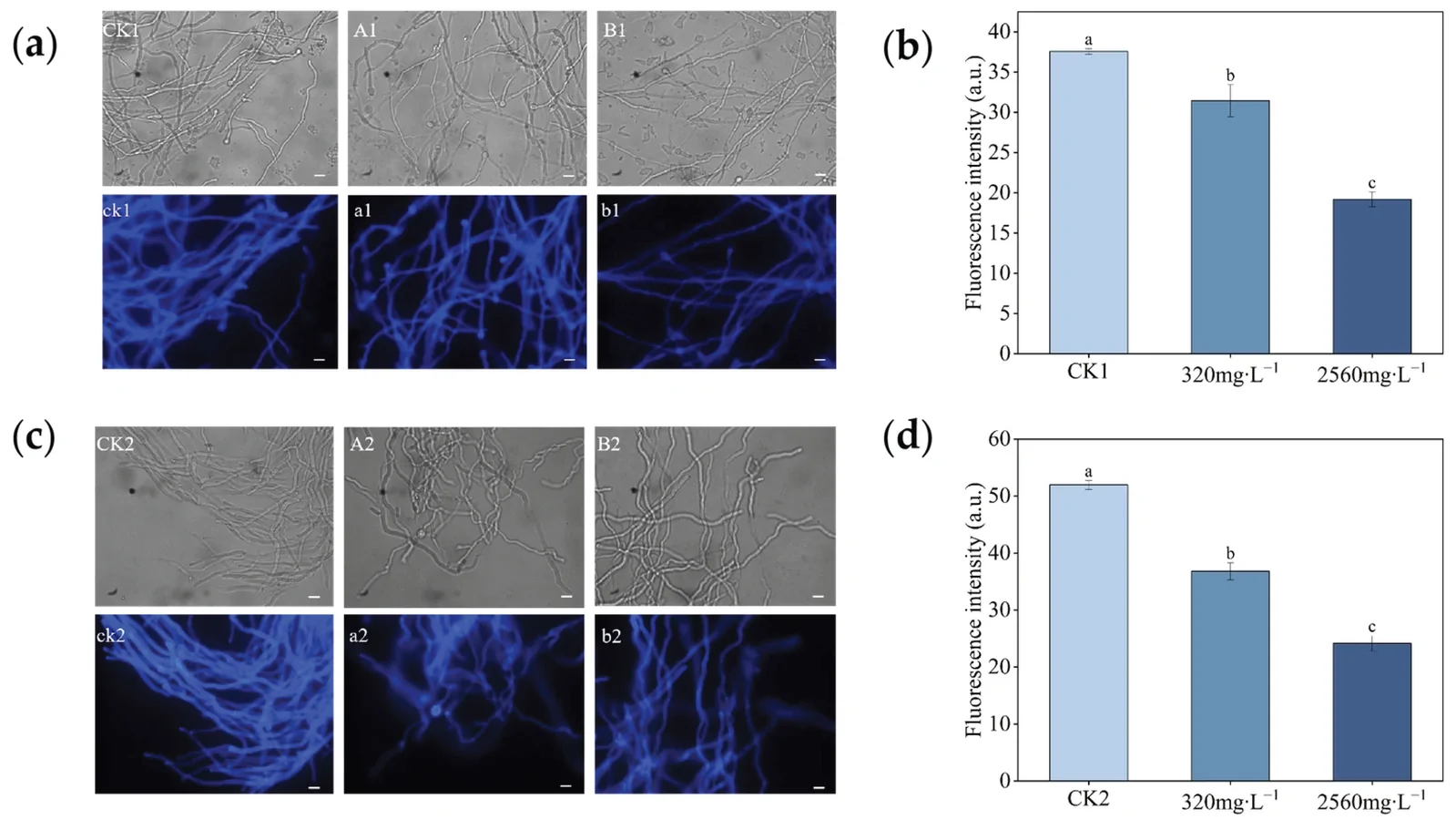
Effects of SeMP treatment on the hyphal cell walls and corresponding fluorescence intensity of *P. italicum* and *P. digitatum*. (**a**,**c**) Bright-field and corresponding fluorescence images of *P. italicum* and *P. digitatum* hyphae treated with 0, 320, and 2560 mg·L^−1^ SeMPs, respectively; (**b**,**d**) quantitative analysis of cell wall fluorescence intensity in *P. italicum* and *P. digitatum*, respectively. Different lowercase letters above the bars indicate significant differences among treatments (*p* < 0.05). Scale bar = 100 μm.

To evaluate SeMPs-associated changes in the hyphal cell wall of *P. italicum* and *P. digitatum*, Calcofluor White (CFW) staining was used to examine changes in the fluorescence intensity and distribution of CFW-binding cell-wall components. As shown in Figure 6a,c, untreated hyphae of both pathogens exhibited clear, continuous, and relatively strong blue fluorescence along the cell wall, reflecting the normal distribution of CFW-binding polysaccharide components. Following treatment with 320 mg·L^−1^ SeMPs, CFW fluorescence decreased in both pathogens, and a further reduction was observed at 2560 mg·L^−1^. These changes in fluorescence intensity and distribution indicate that SeMP treatment altered CFW-binding cell-wall components and are consistent with disturbance of hyphal cell-wall integrity.

Quantitative fluorescence analysis (Figure 6b,d) further showed that CFW fluorescence intensity decreased significantly with increasing SeMP concentration in both pathogens, with significant differences among the treatment groups (*p* < 0.05). The lowest fluorescence intensity was observed at 2560 mg·L^−1^. Consistent decreases in CFW fluorescence intensity were also observed at the intermediate concentrations of 640 and 1280 mg·L^−1^, as shown in Figure S3, further supporting an overall concentration-dependent response. Taken together, the qualitative and quantitative CFW staining results demonstrate concentration-dependent changes in the fluorescence intensity and distribution of CFW-binding cell-wall components in both fungi, supporting SeMP-associated perturbation of hyphal cell-wall integrity.

### 3.7. Effect of SeMP Treatment on Reactive Oxygen Species

**Figure 7:**
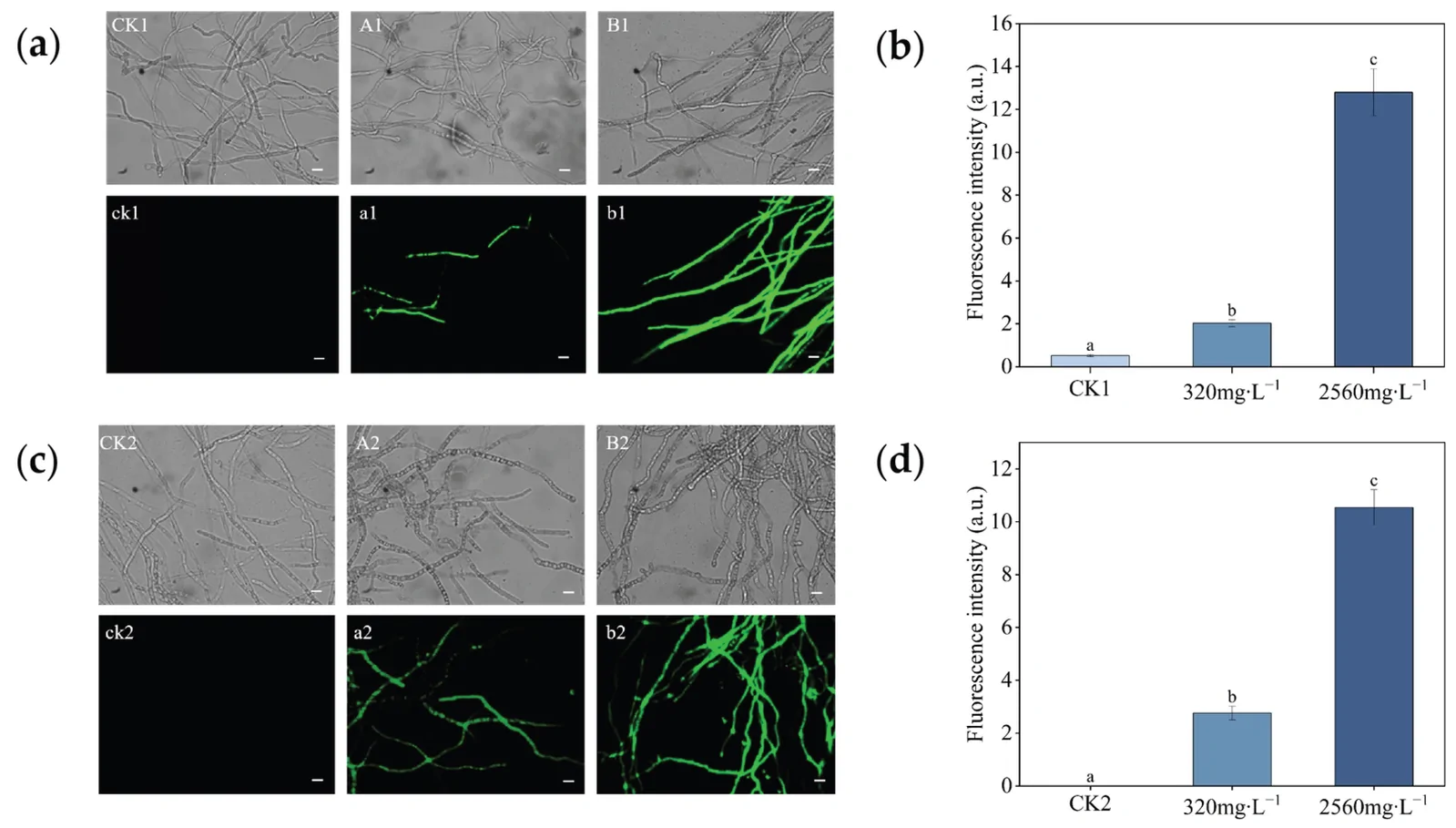
Effects of SeMP treatment on intracellular reactive oxygen species (ROS) accumulation and fluorescence intensity in *P. italicum* and *P. digitatum* hyphae. (**a**,**c**) Bright-field images and corresponding ROS fluorescence images of *P. italicum* and *P. digitatum* hyphae treated with 0, 320, and 2560 mg·L^−1^ SeMPs, respectively; (**b**,**d**) quantitative analysis of intracellular ROS fluorescence intensity in *P. italicum* and *P. digitatum*, respectively. Different lowercase letters above the bars indicate significant differences among treatments (*p* < 0.05). Scale bar = 100 μm.

To evaluate the effect of SeMPs on intracellular reactive oxygen species (ROS) accumulation in *P. italicum* and *P. digitatum* hyphae, ROS levels were assessed using the DCFH-DA fluorescent probe. As shown in Figure 7a,c, no obvious green fluorescence was observed in the hyphae of either pathogen in the control groups, indicating that intracellular ROS levels remained low under normal conditions. Following treatment with 320 mg·L^−1^ SeMPs, both pathogens exhibited weak and discontinuous green fluorescence. When the SeMP concentration was increased to 2560 mg·L^−1^, the green fluorescence signals were markedly enhanced, with a substantial increase in the number of positively stained hyphae and strong fluorescence observed in most hyphae.

Quantitative analysis of fluorescence intensity (Figure 7b,d) further showed that intracellular ROS fluorescence increased significantly in both *P. italicum* and *P. digitatum* with increasing SeMP concentration, with significant differences among all treatment groups (*p* < 0.05). The highest fluorescence intensity was observed at 2560 mg·L^−1^. These quantitative results were consistent with the fluorescence microscopy observations, indicating that SeMPs induced intracellular ROS accumulation in both pathogens in a concentration-dependent manner. Notably, under the high SeMP concentration of 2560 mg·L^−1^, *P. italicum* exhibited stronger ROS signals than *P. digitatum*, indicating species-dependent differences in oxidative responses. However, the physiological basis for this difference remains unclear. Differences in antioxidant capacity or other species-specific cellular characteristics may contribute to the observed variation in SeMP susceptibility, but these possibilities were not directly examined in the present study. A similar concentration-dependent increase in ROS fluorescence was also observed at 640 and 1280 mg·L^−1^ SeMPs, as shown in Figure S4.

### 3.8. Effect of SeMP Treatment on Extracellular Conductivity

**Figure 8:**
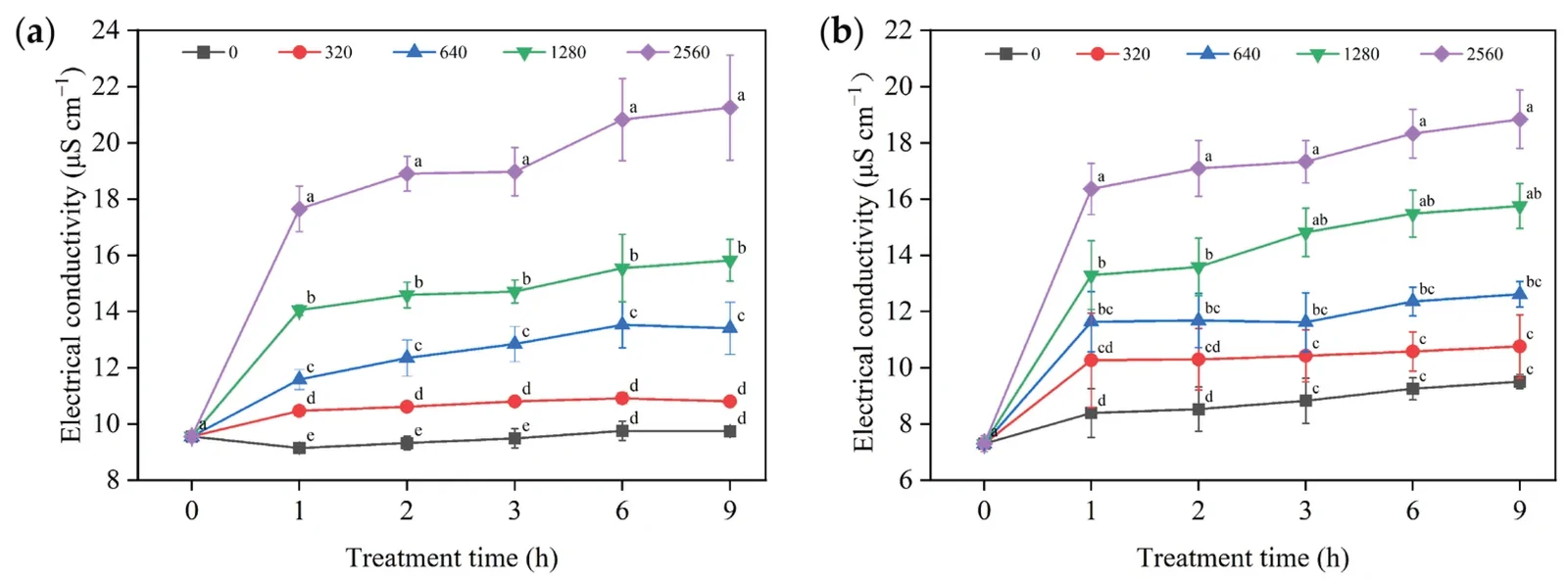
Extracellular conductivity of *P. italicum* and *P. digitatum* after SeMP treatment. (**a**) *P. italicum*; (**b**) *P. digitatum*. Hyphae were treated with SeMPs at 0, 320, 640, 1280, and 2560 mg·L^−1^, and extracellular conductivity was measured at 0, 1, 2, 3, 6, and 9 h post-treatment. Data are expressed as µS cm^−1^. Lines sharing the same letter are not significantly different.

As shown in Figure 8, SeMP treatment caused the extracellular conductivity of both *P. italicum* and *P. digitatum* to increase in a generally time- and concentration-dependent manner. The conductivity of all treatment groups of both pathogens increased rapidly within 1 h after treatment and then rose slowly.

For *P. italicum* (Figure 8a), the 2560 mg·L^−1^ treatment group maintained the highest extracellular conductivity throughout, reaching 21.2 ± 1.87 μS cm^−1^ at 9 h, compared with 9.8 ± 0.078 μS cm^−1^ for the control; the 1280 mg·L^−1^ group ranked next. For *P. digitatum* (Figure 8b), the 2560 and 1280 mg·L^−1^ groups showed the most obvious increases, reaching 18.8 ± 1.03 and 15.8 ± 0.79 μS cm^−1^ at 9 h, respectively, whereas the low-concentration groups changed relatively little.

The above results indicate that high SeMP concentrations can significantly increase the cell membrane permeability of both *Penicillium* species. The rapid rise in conductivity suggests that SeMPs can disrupt membrane function and cause efflux of intracellular electrolytes at the early stage of treatment. In addition, the greater increase in extracellular conductivity observed in *P. italicum* at high SeMP concentrations was consistent with its stronger SYTOX Green fluorescence and higher ROS accumulation, indicating a greater overall sensitivity to SeMP-associated cellular stress than *P. digitatum*.

### 3.9. Effect of SeMP Treatment on Nucleic Acid and Protein Leakage

**Figure 9:**
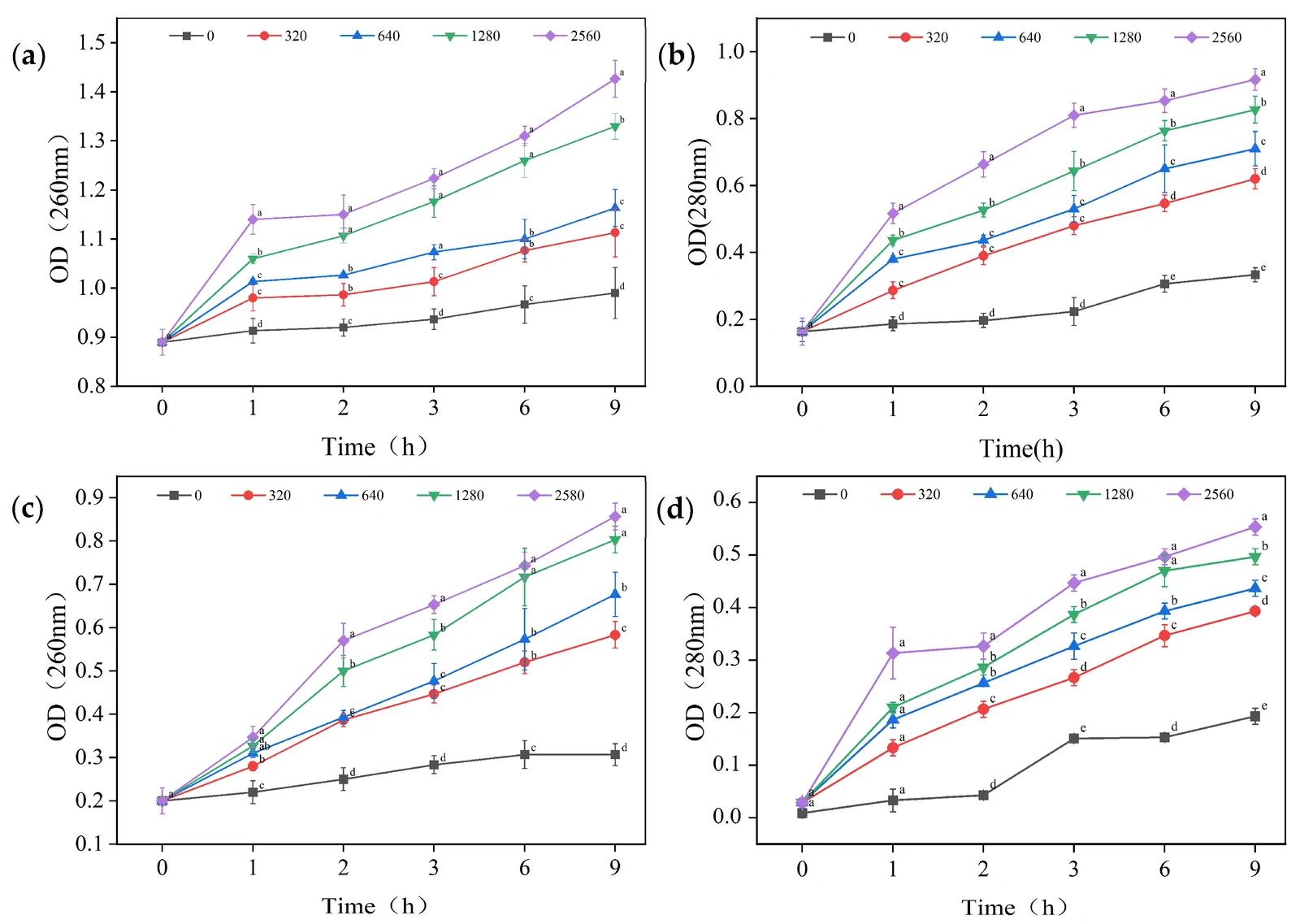
Nucleic acid and protein leakage from *Penicillium italicum* and *P. digitatum* after SeMP treatment. (**a**,**b**) *P. italicum*: (**a**) nucleic acid leakage (OD260), (**b**) protein leakage (OD280); (**c**,**d**) *P. digitatum*: (**c**) nucleic acid leakage (OD260), (**d**) protein leakage (OD280). Hyphae were treated with SeMPs at 0, 320, 640, 1280, and 2560 mg·L^−1^, and the extracellular supernatants were sampled at 0, 1, 2, 3, 6, and 9 h post-treatment for OD260 and OD280 measurement. Lines sharing the same letter are not significantly different.

As shown in Figure 9, SeMP treatment caused the OD260 and OD280 values of the extracellular supernatants of both *P. italicum* and *P. digitatum* to increase overall with increasing treatment concentration and prolonged treatment time, indicating that nucleic acid and protein leakage from both pathogens was promoted. Both indicators rose rapidly in the early stage of treatment and then gradually slowed, showing clear concentration- and time-dependence.

For *P. italicum*, high SeMP concentrations produced the most pronounced promotion of nucleic acid and protein leakage (Figure 9a,b). The 1280 and 2560 mg·L^−1^ groups showed significantly higher OD260 and OD280 values than the low-concentration and control groups at most measurement time points (*p* < 0.05), with the 2560 mg·L^−1^ group consistently maintaining a high level. After 9 h of treatment, the OD260 and OD280 values of this group reached 1.43 ± 0.038 and 0.92 ± 0.032, respectively, whereas those of the control were 0.99 ± 0.052 and 0.33 ± 0.021. Treatment at 320 and 640 mg·L^−1^ also increased nucleic acid and protein leakage, but to a smaller extent, and some adjacent concentration groups showed no significant difference at individual time points (*p* > 0.05).

*P. digitatum* showed a similar trend (Figure 9c,d). With increasing SeMP concentration, the extracellular OD260 and OD280 values continued to increase, with the 1280 and 2560 mg·L^−1^ groups showing the most pronounced promotion. After 9 h of treatment, the OD260 and OD280 values of the 2560 mg·L^−1^ group reached 0.86 ± 0.031 and 0.55 ± 0.015, respectively, whereas those of the control were only 0.31 ± 0.025 and 0.19 ± 0.015.

In summary, SeMPs can induce the release of intracellular nucleic acids and proteins to the extracellular space in both *Penicillium* species, and the effect intensified with concentration and time.

## 4. Discussion

Microorganism-mediated synthesis has attracted increasing attention as a green route for producing biologically active particulate materials, particularly for antimicrobial and food-related applications [31]. Microbial reduction in selenite or selenate is generally regarded as a detoxification and transformation process that results in the formation of selenium particles [32,33]. Previous studies have demonstrated that various bacteria, including *Bacillus*, *Pseudomonas*, *Streptomyces*, and *Lysinibacillus* spp., can produce selenium particles with strain-dependent differences in size and morphology [32,33,34,35,36,37,38,39]. In the present study, *L. sphaericus* N30 produced predominantly spherical selenium-rich particles, and DLS analysis showed a Z-average hydrodynamic diameter of 656.5 nm with a PDI of 0.274. Accordingly, these particles were classified as selenium microparticles (SeMPs) rather than conventional SeNPs. Importantly, selenium particles in this size range are not unprecedented: Tuyen et al. [17] reported spherical SeMPs with an average diameter of approximately 475 nm, which exhibited antibacterial and antifungal activities, while Dat et al. [16] also synthesized SeMPs and demonstrated their antimicrobial potential; more recently, submicron-sized selenium particles have been explicitly reported as biologically active selenium materials [40]. The relatively larger size of SeMPs compared with many previously reported bacterial SeNPs may be associated with strain-specific selenium reduction kinetics, culture conditions, and interactions with extracellular biomolecules during particle formation [32,37]. Therefore, the 656.5-nm hydrodynamic size of SeMPs should be regarded as a characteristic of the specific biogenic system rather than an abnormal particle size, and previous evidence that several-hundred-nanometer SeMPs retain antimicrobial activity supports the further evaluation of their antifungal efficacy in the present study.

The efficacy of selenium-based materials against postharvest citrus diseases is strongly influenced by the applied concentration, as sufficient exposure at the infection site is likely required to effectively restrict pathogen establishment and subsequent colonization. Previous studies have demonstrated effective control of citrus green mold using SeNPs-containing composite formulations. Salem et al. reported complete suppression of *P. digitatum* on orange fruit using an NCT/PPE/SeNPs coating, whereas Tayel et al. achieved effective protection of infected lemons using a chitosan/fenugreek mucilage/SeNPs nanocomposite [41,42]. However, these formulations combined nanoscale selenium with chitosan and plant-derived antimicrobial components, and their protective effects may therefore reflect the combined contribution of multiple active constituents. In the present study, N30-derived SeMPs also exhibited concentration-dependent suppression of both *P. italicum* and *P. digitatum* without the addition of other antimicrobial components. This observation, together with previous reports of antimicrobial activity for SeMPs in the several-hundred-nanometer range [16,17], suggests that selenium particles of this size can retain antifungal potential without necessarily being incorporated into nanoscale composite formulations. Their efficacy may therefore depend not only on particle size, but also on the applied concentration and the extent of particle–pathogen interaction at the infection site.

The cell membrane is an important barrier for maintaining intracellular homeostasis and normal physiological functions in pathogenic fungi, including solute transport, signal transduction, and osmotic regulation. Disruption of membrane integrity, oxidative stress, and leakage of intracellular constituents are frequently associated with the antimicrobial effects of particulate materials [12]. In the present study, SeMP treatment induced a concentration-dependent increase in intracellular ROS accumulation in both *P. italicum* and *P. digitatum*, accompanied by pronounced alterations in hyphal morphology and membrane integrity. SEM observations revealed hyphal shrinkage, surface depression, and local disruption, while enhanced SYTOX Green fluorescence indicated increased membrane permeability. The consistently stronger SYTOX Green responses observed in *P. italicum* at 640, 1280, and 2560 mg·L^−1^ were consistent with its greater susceptibility to SeMP treatment. In parallel, increases in extracellular conductivity and in the extracellular absorbance at 260 and 280 nm were observed during SeMP exposure, indicating enhanced leakage of intracellular electrolytes and nucleic-acid- and protein-associated UV-absorbing constituents, respectively. The concurrence of ROS accumulation, membrane permeabilization, and intracellular leakage suggests a close association between oxidative stress and membrane dysfunction during SeMP exposure, although their causal and temporal relationship cannot be established from the present data. This interpretation is supported by observations in *P. digitatum* that increasing oxidative stress is accompanied by increased ROS production, lipid peroxidation, and membrane permeability [43]. Related studies with nanoscale selenium provide additional mechanistic parallels: SeNPs exposure has been associated with increased membrane permeability in *P. capsici* [44], severe hyphal membrane disruption in *P. italicum* [45], and concurrent oxidative stress and structural damage in phytopathogenic fungi [46]. Moreover, SeNPs-induced ROS accumulation has been linked to membrane damage and subsequent leakage of intracellular constituents in microbial systems [47,48]. These observations suggest that oxidative imbalance and loss of membrane homeostasis may represent interconnected cellular responses to selenium-particle exposure, rather than independent consequences of treatment.

From the perspective of commercial translation, the current SeMP strategy should be regarded as a laboratory-scale proof of concept rather than a technology ready for near-term implementation in citrus packinghouses. The multistep production process, involving microbial cultivation, cell disruption, SDS-assisted washing, organic-solvent treatment, dialysis, and freeze-drying, may limit large-scale production, particularly because commercial dipping would require substantial volumes of treatment suspension and therefore large amounts of selenium material. Moreover, although selenium is an essential trace element, excessive exposure may pose safety concerns, making both fruit quality and residue safety critical considerations for practical application. Under the conditions tested in this study, no obvious visible peel injury or abnormal discoloration was observed in the representative fruit images, even at the higher SeMP concentrations; however, fruit-quality attributes were not quantitatively evaluated. Therefore, potential effects on peel color, firmness, soluble solids, titratable acidity, sensory quality, and other physiological characteristics remain to be systematically assessed. In addition, selenium residues in the peel and internal fruit tissues were not quantified, and the retention, migration, chemical form, and resulting dietary exposure to selenium therefore remain unknown. Future work should focus on simplifying and scaling up production, improving product stability and batch consistency, reducing material consumption, developing lower-volume application strategies, and establishing comprehensive quality and food-safety assessments. Accordingly, substantial process optimization and safety validation are still required before this approach can be realistically translated to commercial postharvest use.

## 5. Conclusions

This study demonstrates that biogenic selenium microparticles derived from *Lysinibacillus sphaericus* N30 exhibit antifungal activity against *Penicillium italicum* and *P. digitatum* and can suppress the development of citrus postharvest blue and green molds under controlled experimental conditions. The antifungal effects of N30-derived SeMPs were associated with pronounced alterations in hyphal morphology and cell-wall-associated structures, increased membrane permeability, intracellular ROS accumulation, enhanced extracellular conductivity, and leakage of intracellular constituents, indicating that membrane dysfunction and oxidative stress are important cellular responses to SeMP exposure. Differences in susceptibility between *P. italicum* and *P. digitatum* further suggest species-dependent responses to the selenium material, although the causal relationships among oxidative stress, membrane perturbation, and intracellular leakage remain to be clarified. Overall, these findings provide laboratory-scale proof of concept for the potential use of N30-derived SeMPs as a postharvest antifungal material for citrus. Further studies are required to optimize production and application strategies and to systematically evaluate fruit quality, selenium residues and migration, toxicological safety, and regulatory compliance before commercial postharvest application can be considered.

## Data Availability

The original contributions presented in this study are included in the article/Supplementary Materials. Further inquiries can be directed to the corresponding author.

## Supplementary Materials

The following are available online at https://www.mdpi.com/article/10.3390/foods15183249/s1, Figure S1: Multilocus phylogenetic tree of *Penicillium*strains based on concatenated ITS, BenA, CaM, and RPB2 sequences. *Penicillium italicum*GJPI002 and *P. digitatum* GJPD001 used in this study are indicated by red circles; Figure S2: Effects of SeMP treatment on hyphal membrane integrity and fluorescence intensity in *P. italicum* and *P. digitatum*. (**a**,**c**) Bright-field and corresponding fluorescence images of *P. italicum* and *P. digitatum* hyphae treated with 0, 640, and 1280 mg·L^−1^ SeMPs, respectively; (**b**,**d**) quantitative analysis of hyphal membrane fluorescence intensity in *P. italicum* and *P. digitatum*, respectively. Different lowercase letters above the bars indicate significant differences among treatments (*p* < 0.05). Scale bar = 100 μm; Figure S3: Effects of SeMP treatment on hyphal cell walls and corresponding fluorescence intensity in *P. italicum* and *P. digitatum*. (**a**,**c**) Bright-field and corresponding fluorescence images of *P. italicum* and *P. digitatum* hyphae treated with 0, 640, and 1280 mg·L^−1^ SeMPs, respectively; (**b**,**d**) quantitative analysis of cell wall fluorescence intensity in *P. italicum* and *P. digitatum*, respectively. Different lowercase letters above the bars indicate significant differences among treatments (*p* < 0.05). Scale bar = 100 μm; Figure S4: Effects of SeMP treatment on intracellular reactive oxygen species (ROS) accumulation and fluorescence intensity in *P. italicum* and *P. digitatum* hyphae. (**a**,**c**) Bright-field images and corresponding ROS fluorescence images of *P. italicum* and *P. digitatum* hyphae treated with 0, 640, and 1280 mg·L^−1^ SeMPs, respectively; (**b**,**d**) quantitative analysis of intracellular ROS fluorescence intensity in *P. italicum* and *P. digitatum*, respectively. Different lowercase letters above the bars indicate significant differences among treatments (*p* < 0.05). Scale bar = 100 μm.
