# Supplementary material for: Antifungal Efficacy of Selenium Microparticles Biosynthesized by Lysinibacillus sphaericus Against Citrus Postharvest Blue and Green Molds: Mechanistic Insights
Source: Foods. 2026 Sep 14;15(18):3249. doi: 10.3390/foods15183249 (PMC13606604; doi:10.3390/foods15183249)
Supplement: Supplementary file 1 [file foods-15-03249-s001.zip › foods-4536107-supplementary.pdf]

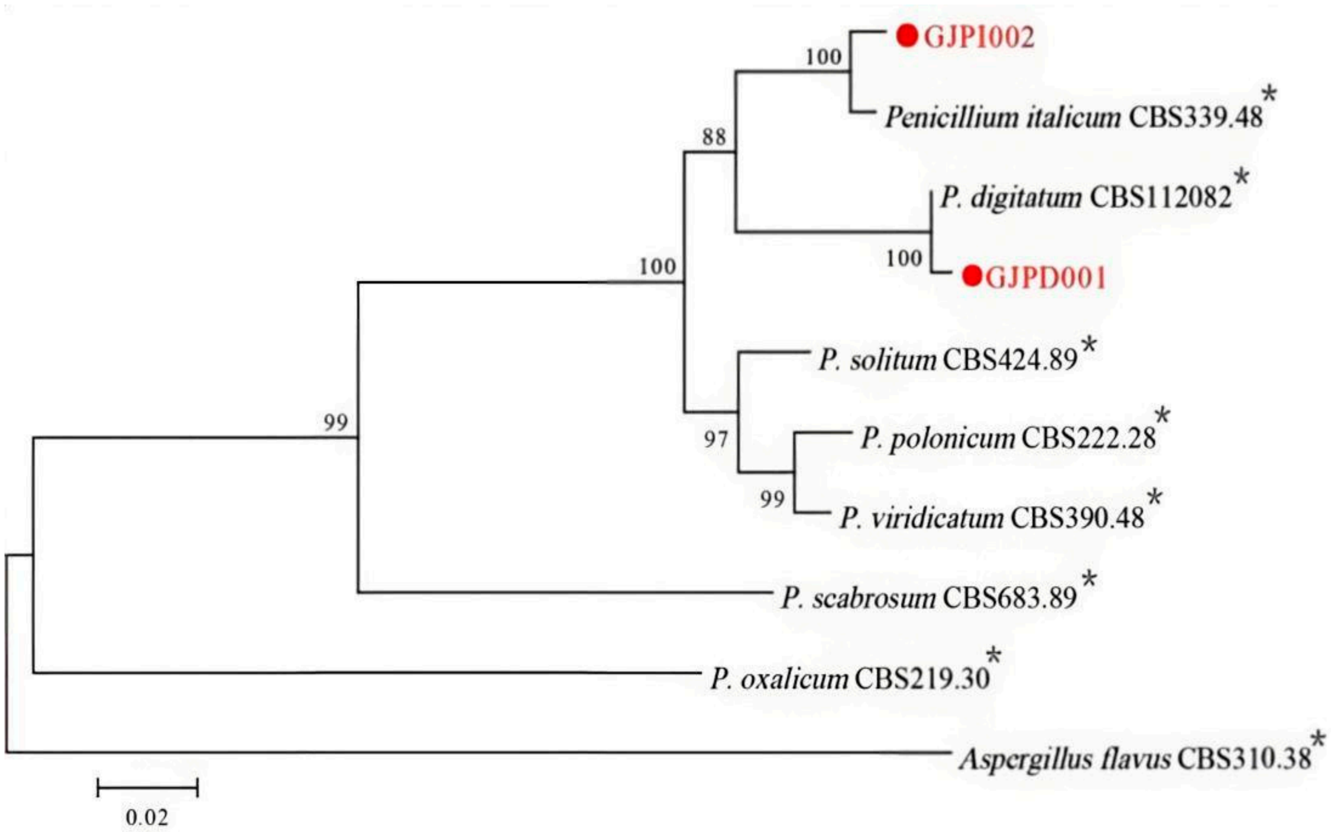

Figure S1. Multilocus phylogenetic tree of Penicillium strains based on concatenated ITS, BenA, CaM, and RPB2 sequences. *Penicillium italicum* GJPI002 and *P. digitatum* GJPD001 used in this study are indicated by red circles.

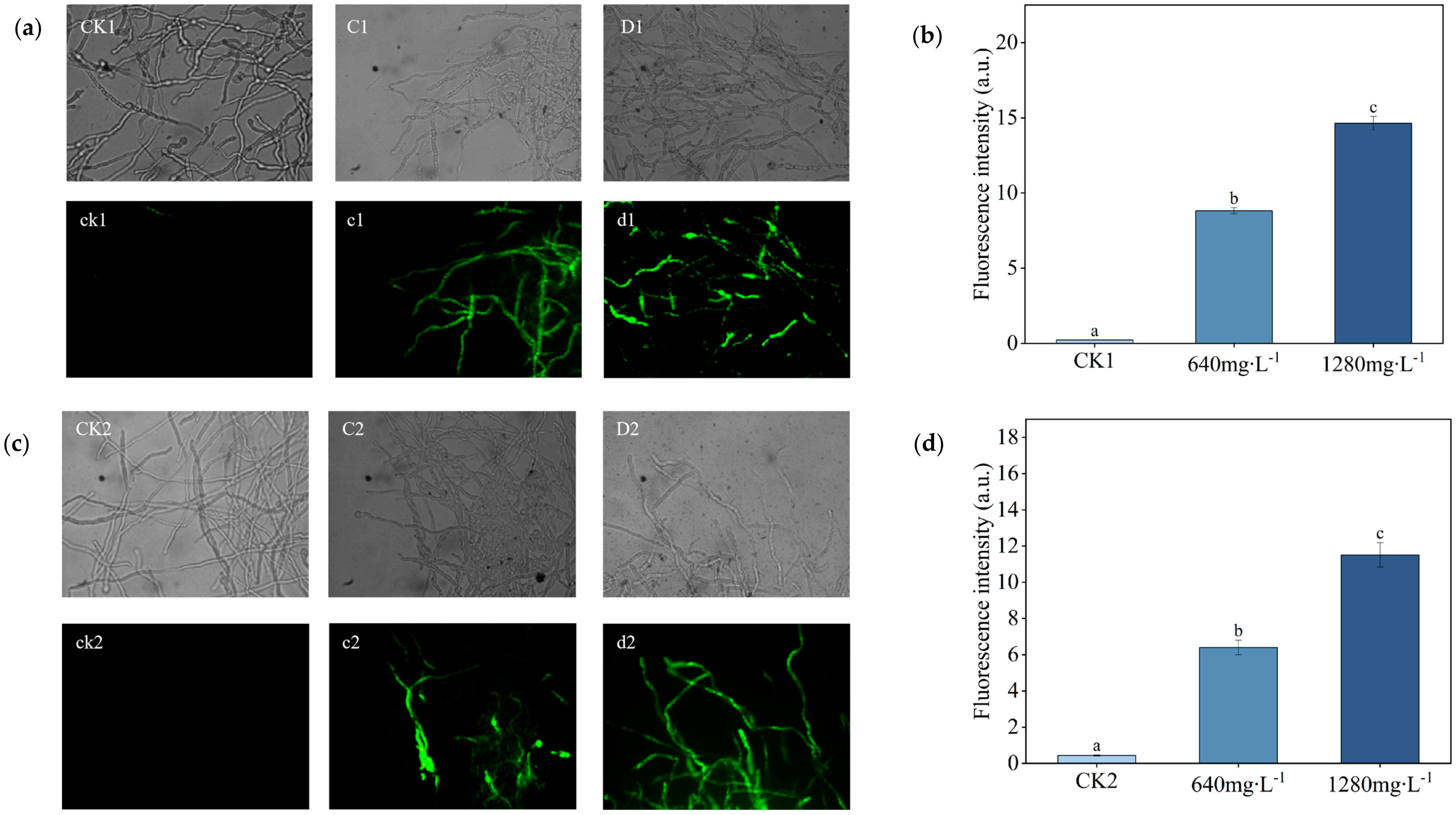

Figure S2. Effects of SeMP treatment on hyphal membrane integrity and fluorescence intensity in *P. italicum* and *P. digitatum*. (**a**,**c**) Bright-field and corresponding fluorescence images of *P. italicum* and *P. digitatum* hyphae treated with 0, 640, and 1280 mg·L^{-1} SeMPs, respectively; (**b**,**d**) quantitative analysis of hyphal membrane fluorescence intensity in *P. italicum* and *P. digitatum*, respectively. Different lowercase letters above the bars indicate significant differences among treatments ($p < 0.05$). Scale bar = 100 μm.

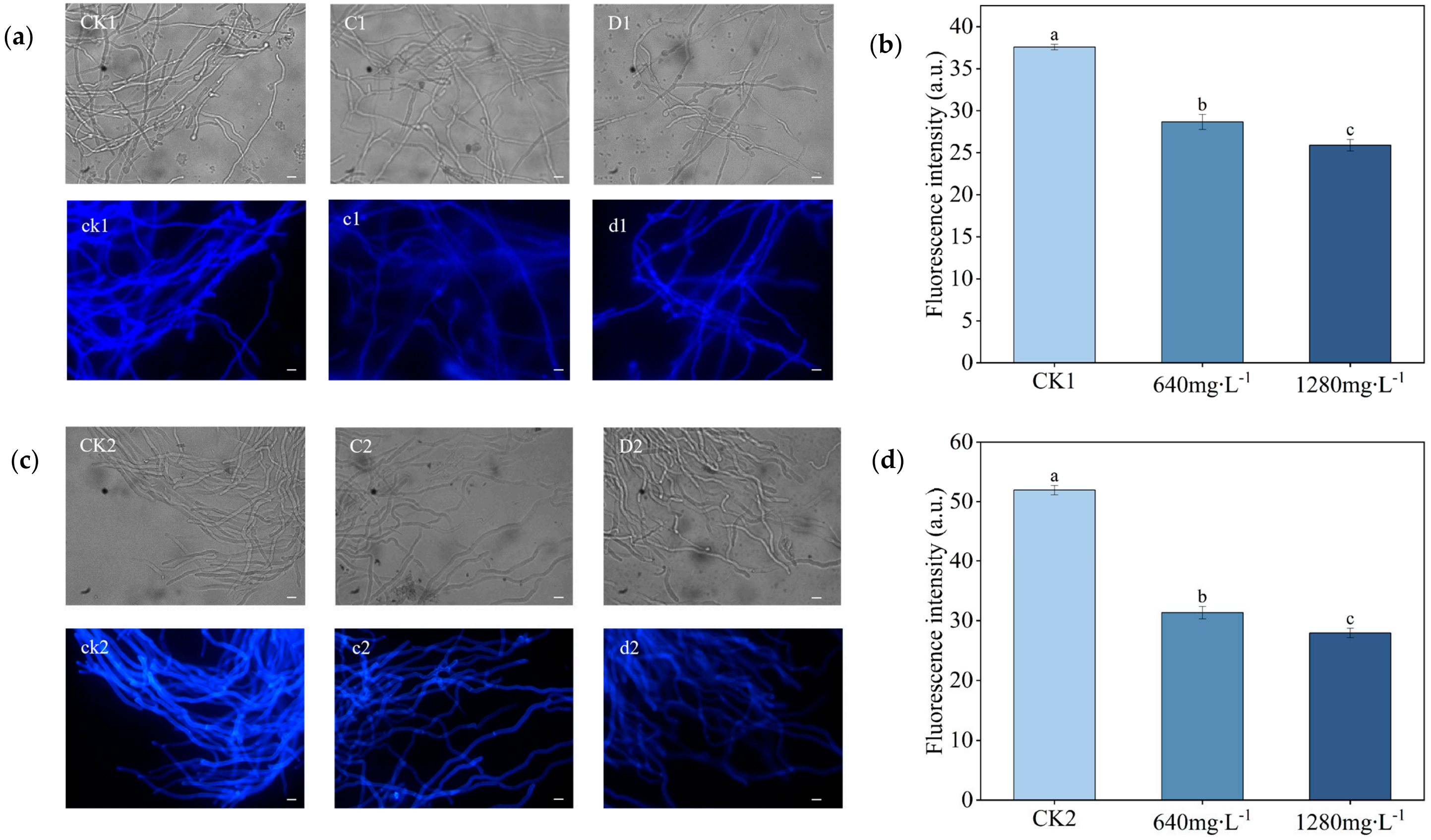

Figure S3. Effects of SeMP treatment on hyphal cell walls and corresponding fluorescence intensity in *P. italicum* and *P. digitatum*. (**a**,**c**) Bright-field and corresponding fluorescence images of *P. italicum* and *P. digitatum* hyphae treated with 0, 640, and 1280 mg·L^{-1} SeMPs, respectively; (**b**,**d**) quantitative analysis of cell wall fluorescence intensity in *P. italicum* and *P. digitatum*, respectively. Different lowercase letters above the bars indicate significant differences among treatments ($p < 0.05$). Scale bar = 100 μm.

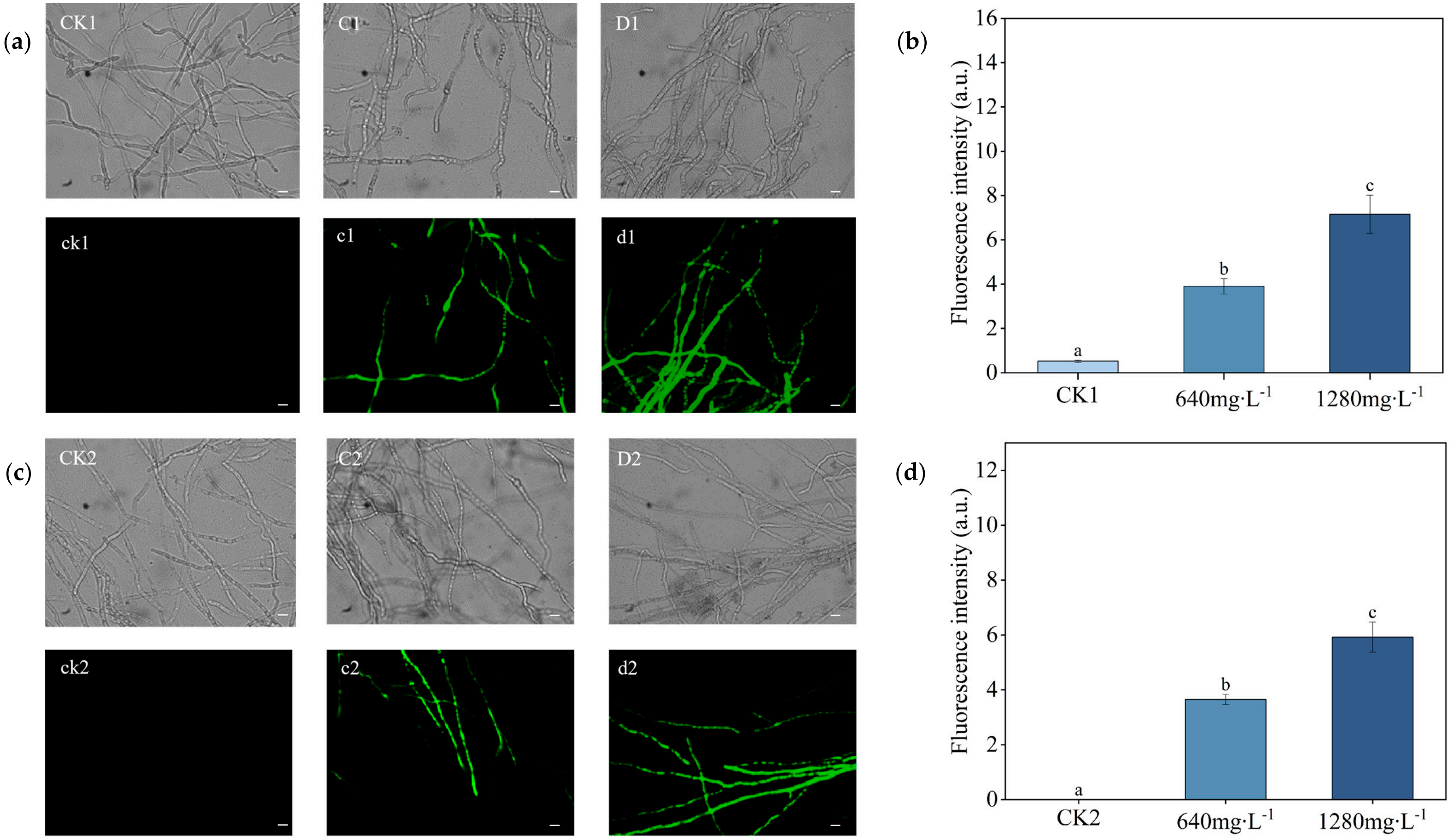

Figure S4. Effects of SeMP treatment on intracellular reactive oxygen species (ROS) accumulation and fluorescence intensity in *P. italicum* and *P. digitatum* hyphae. (**a**,**c**) Bright-field images and corresponding ROS fluorescence images of *P. italicum* and *P. digitatum* hyphae treated with 0, 640, and 1280 mg·L^{-1} SeMPs, respectively; (**b**,**d**) quantitative analysis of intracellular ROS fluorescence intensity in *P. italicum* and *P. digitatum*, respectively. Different lowercase letters above the bars indicate significant differences among treatments ($p < 0.05$). Scale bar = 100 μm.
